# Evaluating Illumina-, Nanopore-, and PacBio-based genome assembly strategies with the bald notothen, *Trematomus borchgrevinki*

**DOI:** 10.1093/g3journal/jkac192

**Published:** 2022-07-29

**Authors:** Niraj Rayamajhi, Chi-Hing Christina Cheng, Julian M Catchen

**Affiliations:** Department of Evolution, Ecology, and Behavior, University of Illinois, Urbana-Champaign, Champaign, IL 61801, USA; Department of Evolution, Ecology, and Behavior, University of Illinois, Urbana-Champaign, Champaign, IL 61801, USA; Department of Evolution, Ecology, and Behavior, University of Illinois, Urbana-Champaign, Champaign, IL 61801, USA

**Keywords:** genome assembly, k-mer analysis, short-read assembly, long-read assembly, notothenioids

## Abstract

For any genome-based research, a robust genome assembly is required. De novo assembly strategies have evolved with changes in DNA sequencing technologies and have been through at least 3 phases: (1) short-read only, (2) short- and long-read hybrid, and (3) long-read only assemblies. Each of the phases has its own error model. We hypothesized that hidden short-read scaffolding errors and erroneous long-read contigs degrade the quality of short- and long-read hybrid assemblies. We assembled the genome of *Trematomus borchgrevinki* from data generated during each of the 3 phases and assessed the quality problems we encountered. We developed strategies such as k-mer-assembled region replacement, parameter optimization, and long-read sampling to address the error models. We demonstrated that a k-mer-based strategy improved short-read assemblies as measured by Benchmarking Universal Single-Copy Ortholog while mate-pair libraries introduced hidden scaffolding errors and perturbed Benchmarking Universal Single-Copy Ortholog scores. Furthermore, we found that although hybrid assemblies can generate higher contiguity they tend to suffer from lower quality. In addition, we found long-read-only assemblies can be optimized for contiguity by subsampling length-restricted raw reads. Our results indicate that long-read contig assembly is the current best choice and that assemblies from phase I and phase II were of lower quality.

## Introduction

The ultimate goal of genome sequencing is to connect the genome to phenotypes of interest. Genome sequencing can be used for the identification of rare variants associated with common human disease (Cirulli and Goldstein 2010), genes associated with agronomically important traits (Tao *et al.* 2019; Li *et al.* 2021), and structural variations potentially associated with adaptation to a novel environment (Kim *et al.* 2019). Sequencing technology has advanced enormously since its early implementation by the human genome project (HGP), launched in 1990 (Levy and Myers 2016). During the HGP, high-quality genome assemblies were generated by sequencing large insert size clones of human chromosomes using an automated Sanger sequencing approach, referred to as first-generation sequencing (Lander *et al.* 2001). However, while Sanger sequencing offered good read accuracy and approximately 1-kb read lengths, this method was expensive, laborious, and low throughput (Metzker 2005; Heather and Chain 2016).

With the advent of massively parallel, second-generation sequencing, the shortcomings of the Sanger strategy were bridged (Heather and Chain 2016), providing for the expansion and democratization of sequencing techniques (Rothberg and Leamon 2008) and a blooming of projects (Liao *et al.* 2019). However, second-generation sequencing reads were much shorter relative to Sanger sequencing (Schatz *et al.* 2010), which precluded resolving repeats longer than the insert size of the sequenced molecules (Alkan et al. 2011). Although certain molecular methods could extend the insert length (Berglund *et al.* 2011), they brought with them additional analysis challenges (Sahlin et al. 2016). And while the individual nucleotides of short reads have a very high fidelity, with an error rate of less than 1% (Bao and Lan 2017), the assemblies built with short-reads were highly fragmented, consisting of tens of thousands of scaffolds (Rhie *et al.* 2021).

In the recent decade, a third-generation of sequencing technology, long-read sequencing (LRS), including Pacific Biosciences (PacBio) and Oxford Nanopore Technologies (ONT) sequencing, are enabling researchers to generate high-quality, contig-level assemblies (Murigneux *et al.* 2020). LRS technologies can generate reads that are tens of kilobase pairs long. For example, continuous long reads (CLR) sequenced on a PacBio Sequel II machine can achieve a raw N50 length of 30–60 kb and an accuracy of 87–92%. The ONT MinIon/GridION sequencer can produce long and ultra-long reads with an N50 of 10–60 and 100–200 kb, respectively, with an accuracy of 87–98%. Using circular consensus sequencing, PacBio HiFi long-reads yield a reduced N50 of 10–20 kb, but with a significant improvement in accuracy (99%; Logsdon *et al.* 2020).

Furthermore, the long reads from PacBio and ONT can span repetitive regions (Rice and Green 2019), which second-generation short reads could not bridge, including most human genome repeats (Logsdon *et al.* 2020). Consequently, third-generation long reads have enabled genome assemblers to produce less-fragmented genome assemblies (Rice and Green 2019) with few or no gaps.

De novo genome assembly strategies have evolved along with changes in the underlying sequencing technologies resulting in 3 distinct phases: (Phase I) short-read-only, (Phase II) short- and long-read hybrid, and (Phase III) long-read-only assemblies. Phases I and II are now anachronistic strategies whereas the phase III assembly strategy is the current state-of-the-art. While phases I and II assemblies could not achieve chromosome-level results of high fidelity [at least, not without the aid of genomic resources such as very dense genetic maps (Fierst 2015)], phase III assemblies can yield full-length chromosomes in contig form, and scaffolding them—using chromosomal capture methods (Burton *et al.* 2013), optical maps (Leinonen and Salmela 2020), or genetic maps (Kim *et al.* 2019)—can reproduce a proper karyotype (Sedlazeck *et al.* 2018; Rice and Green 2019; Giani *et al.* 2020).

In phase I, short reads were generated primarily from Illumina sequencing platforms at large volume and low cost (with alternative technologies eventually outcompeted by Illumina). To generate contigs, short-read-only de novo genome assemblers used *de Bruijn* (Zerbino and Birney 2008; Compeau *et al.* 2011) or string graph structures (Myers 2005; Simpson and Durbin 2012) based on k-mers extracted from the reads. During the contig assembly process, when repetitive regions in the genome exceed the span of overlapping reads, the contiguity of the assembly breaks (Sullivan *et al.* 2015). While second-generation assemblies are highly accurate at a nucleotide level, they are usually highly fragmented because a significant number of repetitive regions are longer than the insert length of the sequenced molecule (Claros *et al.* 2012; Treangen and Salzberg 2012).

To resolve these repetitive regions, short-read-only assemblers typically used information from mate-pair reads (mapped onto assembled contigs) for ordering, orienting, and linking contigs, i.e. scaffolding. To obtain mate-pair reads, genomic DNA fragments sheared to several chosen lengths [from 2 to 20 kb (Ekblom and Wolf 2014)] are end-biotinylated and circularized to form separate libraries. The circular DNA is sheared again, and the small fragments, consisting of the biotin junction are captured and sequenced to obtain sequences from 2 opposite ends of the original, long DNA fragments. During the scaffolding process, an assembler would use the approximate mate-pair distance to estimate the size of gaps (Ns) within and between contigs (Simpson and Pop 2015). However, mate-pair reads are prone to introducing hidden scaffolding errors by joining distantly related contigs based on the presence of common repeats (Sohn and Nam 2018).

Phase II was marked with the advent of third-generation sequencing platforms, as produced by PacBio and ONT. LRS on early models and chemistries of these platforms was expensive, and data yield was low and laden with errors (10–15% error rate) such as spurious insertions, deletions, and mischaracterized homopolymer runs (Bao and Lan 2017; Salmela *et al.* 2017). In phase II, those long-reads were hybridized with short-read assemblies to increase contiguity (e.g. contig/scaffold N50), in at least 2 ways. The low-coverage, long-read contigs were either merged with high-coverage, short-read contigs with software like quickmerge (Chakraborty *et al.* 2016), or the gaps between and within scaffolds of short-read assemblies were filled with error-corrected long reads using software like PBJELLY (English *et al.* 2012).

Both the merging and gap-filling processes appear to improve contig and scaffold N50, however, the merging process could inflate genome size or duplicate genomic regions in the assembly, which becomes visible when examining the structure of single-copy ortholog genes, with software such as BUSCO (Benchmarking Universal Single-Copy Ortholog; Simão *et al.* 2015). For instance, when low-coverage contigs assembled with long reads are aligned and merged with short-read contigs, merging failure or hidden scaffolding errors can lead to generation of spurious duplicated BUSCO genes. When long reads are aligned to a short-read assembly to fill gaps between contigs, misjoins from mate-pair reads can result in spurious genome size expansion.

Phase III commenced when new iterations of long-read sequencer technology and improved molecular protocols led to less expensive and higher-throughput sequencing runs—for example, PacBio has reduced costs by 2-fold and increased throughput 10-fold (van Dijk *et al.* 2018). In phase III, the large volume of long reads can be used to directly assemble contigs with assemblers such as Falcon (Chin *et al.* 2016), Canu (Koren *et al.* 2017), WTDBG2 (Ruan and Li 2020), or Flye (Kolmogorov *et al.* 2019). In general, phase III has dramatically increased the contiguity of assembly components (Amarasinghe *et al.* 2020). Errors in long reads can be corrected through a nonhybrid approach in which instead of using short reads to correct long reads or contigs, the information from overlapping long reads alone is used (Chen *et al.* 2021)—although such self-error correction processes need higher sequencing coverage (Salmela *et al.* 2017; Zhang *et al.* 2020). However, reads of extreme length (tens of thousands of kilobases) or excessive coverage can still degrade the quality of long-read contig assemblies, potentially due to the presence of chimeric reads (Fichot and Norman 2013; White *et al.* 2017). Tools such as yacrd (Marijon *et al.* 2020) have been developed to identify and filter such chimeric reads to improve assembly contiguity.

For any de novo genome-based research, the challenge is not only to assemble a genome of high contiguity but also with high accuracy and completeness. Critical data analysis is required to obtain such accuracy. It is a common practice to use high values of completeness of BUSCO annotations and contiguity metrics (e.g. N50) as a proxy for quality; however, there is a general lack of critical evaluation of these results in the literature. Furthermore, genomes built using a phase II strategy have been widely reported (Das *et al.* 2020; Moran *et al.* 2020) and practitioners new to genome-scale research may assume such assemblies are of high quality solely based on the apparent high contiguity reported in the study. Thus, a critical retrospection of the accuracy of those assemblies, as well as the technical underpinnings of such results, will be a useful resource for the broader research community.

We hypothesize that when short-read-only assemblies have hidden scaffolding error and when low-coverage long-read contigs are erroneous, the quality of short- and long-read hybrid assemblies degrades. In this study, we assembled the genome of *Trematomus borchgrevinki*, a cold specialized Antarctic notothenioid fish with an estimated genome size of 1.28 Gb (Chen *et al.* 2008), for which we had all 3 phases of assembly data to investigate assembly quality problems. We show what a more in-depth analysis of BUSCO scores can reveal about assembly quality, and we developed strategies such as k-mer-assembled region replacement and parameter optimization to address phases I and II error models, while demonstrating that long-read sampling can be used to optimize phase III assemblies.

## Materials and methods

### Sequencing

High molecular weight (HMW) DNA was extracted from red blood cells of a male and a female specimen of *T.borchgrevinki*, caught from McMurdo Sound (78^o^S), Antarctica. For the male, sequencing libraries were constructed for sequencing on 3 different platforms, Illumina, Oxford Nanopore, and PacBio Sequel II (see Supplementary text for details). For the female sample, sequencing was performed only on PacBio Sequel II.

For Illumina sequencing, 5 libraries (2 whole-genome shotgun libraries and 3 mate-pair libraries) were constructed. Two shotgun libraries were prepared using the Hyper Library construction kit (Kapa Biosystems) with no PCR amplification. For the first and the second libraries, insert size ranges of 400–500 and 700–800 bp fragments, respectively, were selected and sequenced on a single lane of HiSeq2500 to generate 250 and 160 bp paired-end reads, respectively. Three mate-pair libraries with insert size ranges of 2–5, 5–7, and 8–12 kb fragments, were constructed using the Nextera Mate Pair Library Sample prep kit (Illumina) followed by the TrueSeq DNA Sample Prep kit (we will refer to them as the 5, 7, and 12 kb mate-pair libraries subsequently). Each mate-pair library was sequenced on one lane of HiSeq2500 for 160 bp paired-end reads, which we refer to as mate-pair reads when paired-end reads are generated from mate-pair libraries.

For Oxford Nanopore sequencing, 12 libraries were made using the SQK-LSK109 ligation sequencing kit (Oxford Nanopore) to produce 1D reads, and each library was sequenced on one SpotON R9.4.1 FLO-MIN106 flowcell using a GridIONx5 sequencer. For PacBio CLR sequencing, 1 library for the female and 2 libraries for the male were constructed with unsheared HMW DNA based on PacBio recommendations, selecting for final library fragments ≥45 kb in length. The library was sequenced on Sequel II SMRT cells with 40 h of data collection. Illumina and Nanopore sequencing were carried out at the Roy J. Carver Biotechnology Center, University of Illinois Urbana-Champaign, and PacBio CLR sequencing was performed at the Genomics and Cell Characterization Core Facility, University of Oregon.

### Construction and comparison of de novo short-read-only genome assemblies with different k-mer sizes

For each sequenced mate-pair library, the adaptors were removed with NxTrim v0.4.1 (O’Connell *et al.* 2015) and reads with a proper mate-pair orientation were separated from those with unknown orientation using the –justmp and –separate parameters. These mate-pair and paired-end reads were assembled with Meraculous (v2.2.2.5, Chapman *et al.* 2011), which employs a Hamiltonian *de Bruijn* graph framework based on k-mers to produce a de novo genome assembly. The assembly process was independently repeated 5 times, each time employing a different k-mer size (i.e. 51, 61, 71, 81, and 91 bp; SupplementaryFig.1).

These 5 phase I assemblies were named after their respective k-mer sizes, as k51, k61, k71, k81, and k91 respectively. For each assembly, we executed QUAST v4.6.2 (Gurevich *et al.* 2013) to estimate contiguity metrics, and we assessed the completeness of 4,584 single-copy orthologs from Actinopterygii-specific OrthoDB v9 using BUSCO v3.0.2 with the default parameters. BUSCO classifies orthologs as (1) single copy and complete (hereafter complete), (2) complete but duplicated (hereafter duplicated), (3) fragmented, or (4) missing. At its core, BUSCO is a wrapper of 3 bioinformatic tools: TBLASTN (Camacho *et al*. 2009), AUGUSTUS (Keller *et al.* 2011), and HMMER (Eddy 2011).

### Reverse complementation and reassembly of k71 as well as AUGUSTUS parameter changes

During the comparative assessment of completeness among the k51, k61, k71, k81, and k91 assemblies, we observed that a subset of k71 scaffolds containing fragmented BUSCO genes was assembled in the opposite orientation in alternative assemblies and contained complete versions of the same BUSCO genes. To test whether changing the orientation of a scaffold can convert a fragmented BUSCO gene to a complete one, we reverse complemented the k71 scaffolds (revcom-k71) and repeated the BUSCO analysis.

We next tested whether the inclusion of mate-pair data can affect an assembly and influence BUSCO scores by reassembling k71 while varying the number of mate-pair libraries in the assembly. First, only paired-end reads were used for reassembly. Next, 3 mate-pair libraries with insert sizes of 5, 7, and 12 kb were added separately to the paired-end data to produce 3 independent assemblies. In addition, the combination of 2 mate-pair libraries having 5 and 7 kb insert size as well as that of all 3 mate-pair libraries with paired-end data was employed separately for reassembling k71. We also reverse complemented scaffolds of the assemblies generated from paired-end reads and (1) one mate-pair library or (2) 2 mate-pair libraries.

We further re-executed BUSCO on the k71 assembly by changing the internal default BUSCO parameter –singlestrand from false to true. This allows one to find overlapping gene models, i.e. alternative transcripts producing different protein-coding sequences, located on opposite strands (by default BUSCO does not permit overlapping gene models). To validate these findings, we ran BUSCO v5.2.0 on the reference genome assembly of zebrafish, GRCz11 (Ensembl v106) as well as on k71 assembly using OrthoDB v10 in 3 ways. In the first and the second round, –singlestrand parameter was toggled false and then true, respectively. Third, we reverse complemented chromosomes or scaffolds with BUSCO genes that were fragmented in the first round but became complete in the second round.

### A k-mer-based strategy to improve the completeness of BUSCO genes in a short-read assembly

We developed and optimized a k-mer-based strategy to improve the completeness of k71 by writing 2 custom Python scripts, INFO and CONTEX. INFO enumerates the following elements of the BUSCO evaluations: (1) the names of fragmented genes in k71, (2) the enclosing scaffolds for those genes, (3) the start and the end basepair positions of each gene, (4) scaffold names in alternative assemblies (k51, k61, k81, and k91) with a complete gene, (5) the start and end basepair positions of those complete alternative genes, and (6) scaffold sequences from k71 and alternative assemblies.

CONTEX imports the data generated by INFO to improve k71 by translocating complete genes from alternative assemblies using a k-mer-based strategy (SupplementaryFig.2). For each fragmented gene, CONTEX retrieves the k71 scaffold as well as the scaffold with a complete gene from an alternative assembly and syncs their orientation. It then k-merizes the whole k71 scaffold and the flanking sequences of the complete gene from the alternative assembly. Whenever k-mers of the flanking sequences and the whole scaffold match, CONTEX replaces the enclosing contig(s) (SupplementaryFig.2). Additional details are provided in the Supplementary Materialsand Methods. The improved k71 assembly generated by CONTEX was named *cork71*.

### Construction of de novo short- and long-read hybrid genome assemblies

As the *cork71* assembly of *T. borchgrevinki* was still highly fragmented, we employed 2 phase II hybrid genome assembly strategies to increase contiguity. The first strategy involved merging low-coverage, long-read-based contigs with *k71*. In detail, first, the raw Nanopore reads were independently assembled with Canu (v1.8, Koren *et al.* 2017) and WTDBG2 (v2.3, Ruan and Li 2020) assemblers and assessed with QUAST. Since the assembly from WTDBG2 had a higher contig N50 it was chosen for further analysis. However, the error-corrected Nanopore reads that Canu generated were reserved. Next, 2 rounds of polishing were executed on the WTDBG2 assembly with Pilon (v1.23, Walker, *et al.* 2014). In the first round, we only corrected small indels and SNPs using the Illumina 2 × 250 bp reads, whereas in the second round, we also included the 2 × 160 bp mate-pair reads and allowed for local reassembly. Since the second polishing strategy resulted in a higher N50, we proceeded only with this data set, which we named as *corNpor*. The assemblies *corNpor* and *k71* were aligned to each other using the nucmer program from the MUMMER package (v3.1, Kurtz *et al.* 2004). For the alignments, *corNpor* was used as the “reference” whereas *k71* as the “query.” The alignments generated due to repeats and duplicates were filtered out with the MUMMER delta-filter program by manipulating the minimum alignment identity (-i) and minimum length of alignment (-l) parameters, including (1) -i 95 -l 0 (default), (2) -i 95 -l 1,000, (3) -i 95 -l 5,000, and (4) -i 95 -l 10,000. After filtering alignments, finally, we merged the reference *corNpor* and the query *cork71* using quickmerge (v0.3, Chakraborty *et al.* 2016) with parameters -hco 5.0 -c 1.5 -l 803500 -ml 5,000 and 5 independent hybrid assemblies were obtained.

These quickmerge-based hybrid assemblies were named, *mergedA*, *mergedB*, *mergedC*, and *mergedD*, after their respective delta-filter values. The overlapping (OVL) to nonoverlapping (n-OVL) sequence ratio between 2 contigs determines the merging of 2 contigs in quickmerge (see the details on how quickmerge works in SupplementaryFile1). By default, any alignment with an OVL/n-OVL ratio less than 1.5 is not considered for merging. The hybrid assemblies were assessed with BUSCO and QUAST and a comparative analysis was performed to determine the factor(s) contributing additional duplicated BUSCO genes.

### Filling gaps within and between scaffolds of a *phase I* assembly with long-reads

In a second strategy to obtain a phase II assembly, the gaps between and within scaffolds of *k71* were filled using PBJELLY (PBSUITE v15.4; English *et al.* 2012) with the error-corrected long reads. Default parameters were used except in the mapping (–mpqv 40) and assembly stages (changed −1, which means never timeout during local reassembly, to 2, which means timeout in 2 s). This gap-filled, de novo hybrid genome assembly was referred to as *filk71*.

### Construction and optimization of a phase III assembly

To further improve our *T. borchgrevinki* assembly, we generated a phase III assembly using PacBio CLR reads with WTDBG2. A subsampling strategy was developed to improve the contiguity of the long-read-only assembly, through different permutations of minimum and maximum raw read length and total raw read coverage to generate different subsets of CLR reads.

We developed a custom Python program, sample_reads.py, to perform the subsampling: the user supplies an estimate of the genome size, a minimum and maximum read length, a target coverage, and given those parameters, the program will randomly sample reads from the input files until the coverage limit is reached. If the user wishes to reconstruct a sampled set of reads, they may specify the same “random” seed to subsequent executions of the script. Each set of sampled reads was then assembled with WTDBG2 and analyzed with BUSCO and QUAST. One round of polishing was performed in the final assembly with the arrow module in GCpp (v2.0.0 PacBio) and analyzed with BUSCO. Ten random reads with length greater than 45 kb were chosen and aligned to the WTDBG2 assembly using minimap (v2.1; Li 2018) and alignments were analyzed with samtools (v1.12; Li *et al.* 2009) to test if a read was chimeric.

## Results

### Short- and long-read sequence data

The sequencing of Illumina libraries selected for 400–500 and 700–800 bp insert lengths separately generated 344,314,404 (83.57× coverage) and 95,269,368 (14.79×) reads, respectively. Three mate-pair libraries with insert sizes 2–5, 5–7, and 8–12 kb generated 115,968,758 (18.01× coverage), 116,808,220 (18.14×), and 133,442,224 (20.72×) reads, respectively. In addition, Nanopore sequencing generated 3,872,632 reads with a mean and average N50 length of 6.6 and 10.5 kb, respectively, for 24.29 Gb total length (23.58× coverage). The PacBio CLR sequencing from a single SMRT cell generated 118.42 Gb (114.97× coverage) in 7,651,558 reads with a mean and N50 length of 23.7 and 33.4 kb, respectively.

### The k71 assembly showed high scaffold N50 but low completeness of BUSCO genes

Among 5 de novo short-read-only assemblies (k51, k61, k71, k81, and k91) generated with Meraculous, k71 had the highest scaffold N50 (746 kb, SupplementaryTable1; SupplementaryFig.3). However, results from BUSCO analyses showed that the number of single-copy, complete genes was the highest in k51 (4,221), with k71 (4,177) in third place (SupplementaryTable2). In addition, a fraction of BUSCO genes that were fragmented in k71 were complete in other assemblies, specifically 62, 46, 30, and 35 fragmented genes in k71 were found complete in k51, k61, k81, and k91, respectively.

### Reverse complementation, reassembly, and AUGUSTUS parameter modification reclassified BUSCO genes

When all the scaffolds of k71 were reverse complemented, a total of 29 fragmented BUSCO genes were reclassified as complete (SupplementaryTables3and4). These 29 cases of gene reclassification were almost always accompanied by changes in gene lengths; however, the underlying candidate genomic regions (i.e. potential gene locations outlined by the TBLASTN component of BUSCO) remained the same or highly similar. For the 29 reclassified genes, typically, the complete gene versions were shorter in length compared to their fragmented versions, while the start and the end positions of these complete versions were mapped within the boundaries of the originally fragmented version. In rare cases, when the complete version was longer than its fragmented version, the start and the end positions of the candidate gene model mapped to 2 different gene models, which were identified as candidates for the fragmented version (SupplementaryFig.4).

The effect of mate-pair libraries on assembly metrics and BUSCO scores was observed through reassembling k71 and the reverse complemented versions. In general, when one or more mate-pair libraries were added to the paired-end reads of k71, the scaffold N50 increased and the number of scaffolds decreased (SupplementaryTable5). In addition, the number of complete and duplicated BUSCO genes increased whereas the number of fragmented and missing BUSCO genes decreased (SupplementaryTable6). Also, the assembly contiguity and BUSCO score were better when 3 mate-pair libraries were added to paired-end data rather than 1 or 2 mate-pair libraries (SupplementaryTables5and6). However, with further investigation, we found inconsistencies in the status of BUSCO genes across reassembled genomes. For example, when the same set of 29 reclassified BUSCO genes in k71 were scanned across the reassembled genomes, the genes that were complete in one reassembled genome were not always complete across other reassembled genomes (SupplementaryTables7and8). In addition, with the replacement of one mate-pair library of a given insert size with another, or the addition of more mate-pair libraries, when a BUSCO gene converted from fragmented to complete and vice-versa (SupplementaryTable7), the corresponding scaffolds with different complete/fragmented gene status were typically found to be oriented in the opposite direction. Also, for some genes, when these scaffolds with different orientations were manually set to the same direction, the status of the same BUSCO gene in the scaffolds across assemblies became the same (SupplementaryTable9).

Instead of reverse complementing all scaffolds in the k71 assembly or reassembled genomes, when we simply enabled the AUGUSTUS “singlestrand” parameter (see *Materials andMethods*), 26 fragmented versions of the 29 reclassified genes converted into their complete versions. In these 26 cases, 22 and 4 complete BUSCO genes became shorter (SupplementaryFig.5a) and longer (SupplementaryFig. 5b), respectively. These 26 complete versions had the exact same gene length and corresponding protein sequence as those we obtained by reverse complementing the scaffolds.

To ensure our results were not anomalous to our *T. borchgrevinki* genome or the specific set of BUSCO annotations, we repeated the analysis using the model zebrafish genome as well as k71 with BUSCO v5.2.0. We found that 6 and 12 fragmented BUSCO genes in zebrafish and k71, respectively, became complete and their length changed, when “singlestrand” was set as true as well as when chromosomes or scaffolds containing them were manually reverse complemented.

### Contig replacement lowered the number of fragmented BUSCO genes in k71

The CONTEX program identified 79 of 130 BUSCO genes that were fragmented in k71 but complete in at least one of the other assemblies (k51, k61, k71, k81, and k91). Using a k-mer size of 31, CONTEX corrected 39 of the 79 fragmented BUSCO genes resulting in the *cork71* assembly (SupplementaryTable10). Of the remaining 40 genes, 39 genes were not corrected because they could not be translocated between assemblies without causing problems with neighboring genes, or the directionality of scaffolds could not be reliably determined between assemblies, or genes showed inconsistent fragmentation status with a change in scaffold direction (i.e. genes were fragmented in one direction but not in another).

### Phase II assemblies increased contiguity and the number of BUSCO gene duplicates

When comparing the *corNpor* assembly at the nucleotide level using Pilon, the total number of bases confirmed against the Illumina short reads was 84.24%. Compared to the phase I *cork71* assembly, all phase II merged assemblies (*A*, *B*, *C*, and *D*) not only had higher scaffold N50 and fewer gaps (Ns per 100 kb, Table 1) but also a higher number of duplicated BUSCO genes. As a reminder (see *Materials andMethods*), we increased the required minimum alignment length between *cork71* and *corNpor* contigs in each assembly from *mergedA to mergedD*. The duplicates decreased from 172 in *mergedA* to 143 in *mergedB* but increased further in *mergedC* (181) and *mergedD* (212, Table 1; SupplementaryFig.6).

**Table 1.** Summary of genome statistics and BUSCOs specific to Actinopterygii clade for phase I, phase II, and phase III assemblies we assembled.

Assembly	No. of scaf	Scaf N50 (Mb)	Scaf total length (Mb)	N’s per 100 kb	No. of Contigs	Contig N50 (kb)	Total contig length (Mb)	C	CS	CD	F	M	Total genes searched
k71	9,399	0.72	746.02	23,813.61	116,693	5.37	568.36	4,272 (93.2%)	4,177 (91.1%)	95 (2.1%)	130 (2.8%)	182 (4.0%)	4,584
*cork71*	9,399	0.72	746.13	23,818.37	116,706	5.37	568.41	4,312 (94.1%)	4,217 (92.0%)	95 (2.1%)	91 (2.0%)	181 (3.9%)	4,584
*corNpor*	N/A	N/A	N/A	N/A	5,394	807.66	843.87	4,435 (96.8%)	4,322 (94.3%)	113 (2.5%)	43 (0.9%)	106 (2.3%)	4,584
*mergedA*	8,426	1.47	751.63	15,018.08	56,003	1,024.86	638.75	4,298 (93.8%)	4,126 (90.0%)	172 (3.8%)	76 (1.7%)	210 (4.5%)	4,584
*mergedB*	8,654	1.40	752.05	15,351.44	57,113	1,001.96	636.60	4,299 (93.8%)	4,156 (90.7%)	143 (3.1%)	75 (1.6%)	210 (4.6%)	4,584
*mergedC*	9,145	1.22	759.96	17,734.96	70,158	470.71	625.18	4,303 (93.8%)	4,122 (89.9%)	181 (3.9%)	78 (1.7%)	203 (4.5%)	4,584
*mergedD*	9,269	0.94	764.50	20,155.11	86,994	9.76	610.41	4,302 (93.8%)	4,090 (89.2%)	212 (4.6%)	83 (1.8%)	199 (4.4%)	4,584
*filk71*	8,055	0.9	933.94	5,639.23	95,999	14.57	881.28	4,372 (95.4%)	4,267 (93.1%)	105 (2.3%	81 (1.8%)	131 (2.8%)	4,584
WTDBG2^r^*	N/A	N/A	N/A	N/A	10,848	758.71	1098.31	N/A	N/A	N/A	N/A	N/A	
WTDBG2^Sr^*	N/A	N/A	N/A	N/A	4,409	2,962.48	924.00	4205 (91.7%)	4085 (89.1%)	120 (2.6%)	134 (2.9%)	245 (5.4%)	4,584
WTDBG2^Sra^	N/A	N/A	N/A	N/A	4,409	2,964.76	924.72	4426 (96.6%)	4317 (94.2%)	109 (2.4%)	37 (0.8%)	121 (2.6%)	4,584

k71 indicates original, uncorrected de novo short-read-only assembly; *cork71* indicates k71 assembly corrected at BUSCO gene level; *corNpor* indicates contig-level assembly built with corrected Nanopore reads with low coverage; *mergedA*, *mergedB*, *mergedC*, and *mergedD* indicates 4 independent quickmerge-based hybrid assemblies; *filk71* indicates gap-filled k71 with corrected Nanopore-reads.

* Uncorrected assembly.

C, complete; CS, complete and single-copy; CD, complete and duplicated; F, fragmented; M, missing.

WTDBG2^r^* indicates uncorrected long-read only assembly built with raw PacBio data using WTDBG2 assembler.

WTDBG2^Sr^* indicates uncorrected long-read only assembly built with 70 Gb subsampled PacBio data (generated by sampling minimum and maximum read lengths of 10 and 40 kb, respectively) using WTDBG2 assembler.

WTDBG2^Sra^ indicates polished long-read-only assembly built with 70 Gb subsampled PacBio data (generated by sampling minimum and maximum read lengths of 10 and 40 kb, respectively) using WTDBG2 assembler.

By comparing many-to-one alignments between scaffolds of *cork71* (*query*) to contigs in *corNpor* (*reference*), we observed many cases in which erroneous BUSCO gene duplication occurred when at least 2 conditions were met. First, at least one query (e.g. Illumina scaffold-1) was merged with the reference (e.g. Nanopore contig-1) to form a hybrid sequence. Second, at least one other distinct query (e.g. Illumina scaffold-2) failed to merge with the same reference (Nanopore contig-1), but both of them contained the same or similar set of BUSCO genes. When only the first condition was met, gene duplications did not occur. However, when the second condition was satisfied (i.e. when merging failure occurred), the set of BUSCO genes became duplicated as the hybrid sequence—generated from the alignments between the reference (Nanopore contig-1) and the query (Illumina scaffold-1) that merged—and the unmerged query (Illumina scaffold-2) were placed together in the merged assembly. Such failures can occur when the OVL portion of the reference and the query sequences was either low or absent (SupplementaryFig.7).

In addition, we observed numerous cases in which an increase in the stringency of the minimum alignment length parameter reduced or even removed the overlapping portion of the alignment. Moreover, the overall number of alignments with a high alignment percentage decreased with the increase in parameter stringency (SupplementaryFig.8). When the stringency was low, we found a case in which the linear order of alignment fragments was disrupted by the inclusion of small, nonhomologous regions of the query and reference sequence. That, in turn, spuriously changed the start position of the query causing quickmerge to calculate a false high value of n-OVL portion of the alignment. This drastically lowered the OVL/n-OVL ratio (see *Materials andMethods*) to a value less than the merging threshold and resulted in merging failure and duplication of BUSCO genes (SupplementaryFig.9). This error, however, was not observed, when the stringency was high as more small alignments were filtered out.

Comparing many-to-one alignments from *corNpor* back to *cork71*, we identified a case in which each merged assembly (*A*, *B*, *C*, and *D*) had 2 sets of 23 genes (46 in total) that were duplicates of each other—the highest we found. These gene sets were in 2 distinct hybrid sequences clustered in a row. These 2 hybrid sequences had one common corresponding query sequence (a scaffold in *cork71*; SupplementaryFig.10) that contained the 23 complete genes. This common query scaffold mapped to regions in 4 distinct reference sequences (contigs of *corNpor*), one mapped to the distal portion of the common query, a second mapped to the proximal portion, and regions from the remaining 2 references mapped in between. While some of these mappings could be eliminated by changing the alignment stringency parameter, the duplication could not be fully prevented. However, when the common query was manually split into 2 parts by breaking it at a gap located upstream of its portion overlapping to the second reference, the duplicated 23 BUSCO genes converted to single-copy, complete genes, confirming the source of the duplication.

### Gap-filling the short-read assembly with long-reads inflated genome size

As an alternative to creating a phase II assembly using quickmerge, we filled gaps in the *k71* assembly using error-corrected Nanopore reads with PBJELLY, generating the assembly *filk71*. Compared to *k71*, the *filk71* had a higher contig N50 (14 kb) and fewer gaps (Ns per 100 kb; 5.6 kb) as well as a longer total length (187 Mb larger; Table 1). However, we found 28,377 gaps in *filk71* were overfilled by PBJELLY. A gap is overfilled when long reads from either side of a gap extend into the gap from its flanking regions expanding the size of the original gap without closing it (SupplementaryFig.11). From BUSCO, we observed that the number of duplicated genes was higher in *filk71* (2.3%, or 105 genes) than in k71 (2.1%, 95 genes; Table 1) and that 37 complete BUSCO genes in *k71* became duplicated in *filk71.*

### Creating and optimizing a phase III assembly

We found that all assemblies built by subsampling raw PacBio long-reads improved the contiguity metrics compared to those obtained from assembling all raw long reads (Table 1; SupplementaryTables11; SupplementaryFig.12). For example, generating 70× coverage (based on a 1 Gb genome size estimate) using read lengths that ranged from 10–40, 15–40, and 15–45 kb, and assembling each subset of reads increased contig N50 more than 3 times, decreased number of contigs by half, and increased the largest contig length by more than 3.5 Mb compared to assembling all raw reads. We also observed variation in contiguity statistics for genome assemblies built with different sets of subsampled reads that represented the same amount of data. For example, shifting the minimum read length from 10 to 15 kb and the maximum read length from 40 to 45 kb, the amount of coverage was the same (70 Gb); however, the number of contigs increased by 370 and the contig N50 decreased by 0.16 Mb (SupplementaryTable11). Also, we found evidence for chimeras among the longest reads, with one read of length 99,920 bp that aligned to 2 contigs of the WTDBG2 assembly with mapping quality of 60.

## Discussion

Here, we aim to elucidate the common sources of error in 3 distinct phases of genome assembly to yield some useful insights. First, for phase I assembly, although mate-pair reads increase contiguity (e.g. N50), they can inflate or deflate the BUSCO score of gene completeness. Mate-pair libraries of different insert sizes can interfere with each other, and a single best combination of mate-pair library types does not appear to exist in our data. A phase I assembly can be improved using a k-mer-based contig replacement strategy, though inconsistencies in alternative assemblies place limits on its efficacy. Second, for phase II assembly, when merging contigs created from low volume long reads with phase I contigs, the presence of sequence errors or small repeat alignments can quickly degrade the quality of the hybrid assembly. This problem grows as more assemblies are merged and in general, it is essential to optimize the alignment parameters used for the merging process. Furthermore, hidden scaffolding error generated from mate-pair libraries in the phase I assembly will further degrade the quality of hybrid assemblies. A critical analysis of BUSCO scores is necessary to evaluate the quality of any hybrid assembly that appears to have high contiguity. Finally, for phase III assembly, long reads generate highly contiguous assemblies; however, chimeric long reads or excessive coverage can lower the contiguity of the assembly. Sampling long reads can improve the contiguity of the long-read-only contig-level assembly.

### Phase I

#### A single k-mer size cannot produce an optimal assembly, as measured by BUSCO

For our phase I assemblies, the short-read assembly with the highest N50 did not have the highest number of complete BUSCO genes while the number of fragmented BUSCO genes varied among assemblies using different k-mer lengths. These patterns are consistent with what was reported by Moran *et al.* (2020) for 4 phase I assemblies of orange throat darter fish. The authors reported that 4 assemblies built with k-mer sizes 49, 59, 69, and 79 had (1) 4,247, 4,241, 4,233, and 4,219 complete BUSCO genes, respectively, (b) 2.4, 2.2, 2.5, and 2.3 Mb of scaffold N50, and (3) 86, 93, 86, and 91 fragmented BUSCO genes. These results suggest that different regions of the genome would assemble better with different k-mer sizes, due to the interaction of k-mer length, the commonality of those k-mers in the genome, and sequencing coverage.

It is well recognized that having nonoptimal k-mer size affects the contiguity of short-read assemblies. Having a k-mer size that is too large can increase assembly fragmentation as large k-mers tend to have difficulty in finding overlapping, adjacent k-mers resulting in gaps. However, having a small k-mer size can increase misassembly as it favors collapsing repeats (Chikhi and Medvedev 2014), which can result in chimeric joins (while additionally, mate-pair reads can spuriously join genomic regions that are far apart; Treangen and Salzberg 2012). In both cases, the intron/exon structures of genes can be prevented from being properly assembled, as reflected in BUSCO results. While some de novo assemblers attempt to apply different k-mer sizes (e.g. Spades, Bankevich *et al.* 2012), it is in practice a difficult problem and one that has been superseded by newer, phase III approaches.

#### Mate-pairs can inflate or deflate BUSCO scores by generating aberrations in phase I assemblies

We found reverse complementing scaffolds can convert some fragmented BUSCO genes to complete versions and vice-versa, although TBLASTN searches, used by BUSCO to outline genomic regions to annotate, yielded the same candidate gene regions in the forward and reverse complemented scaffolds. This evidence suggests that some complete/fragmented BUSCO genes are aberrations that are only counted when contigs end up being in one particular orientation. Since mate-pair reads determine the orientation of a contig within a wider scaffold, they may be the primary culprit for these types of errors.

Swapping mate-pair libraries in our k71 assembly, we observed that corresponding scaffolds in alternative assemblies that had complete or fragmented versions of the same BUSCO gene typically had different orientations. The same pattern occurred when we increased the number of mate-pair libraries for reassembled genomes, and we found some cases in which manually forcing the scaffold orientation to be in the same direction generated the same gene version in all of them. This means that when mate-pair libraries with different insert sizes are mixed together, they can interfere with each other, and in turn, the completeness of a BUSCO gene can change. As mate-pair reads often lead to misjoins in the scaffolding process due to repeats, we think it is a fundamental nature of genomic repeats—and the inability of short reads to bridge them—that is responsible for the errors. Finally, our comparative analyses indicate that potentially the default “singlestrand” parameter in AUGUSTUS can trigger the misannotation of BUSCO genes, depending upon how mate-pair reads orient the underlying contigs, and consequently can contribute to the generation of annotation aberrations. Researchers involved in the application of BUSCO may benefit from varying this parameter in their own assemblies.

Importantly, with BUSCO, when the underlying assembly changes, the genomic lengths of the corresponding single-copy orthologs can change as well. Our comparative analyses suggest that these changes in the BUSCO gene lengths occur through at least 3 processes. First, the length can decrease due to the splitting of a long gene model in one direction into smaller gene models in the alternative direction (SupplementaryFig.5a). Second, the shift in the start or end position of the gene model can decrease (SupplementaryFig.5a) or increase (SupplementaryFig.5a) length. Third, BUSCO gene length can increase through the combination of smaller gene models (SupplementaryFig.5b). Here, we refer to gene models as alternative transcripts resulting in different protein products from the same underlying gene.

#### No combination of mate-pair libraries can be considered better than another for assembly optimization

When we observed 29 BUSCO genes that were fragmented in *k71* but complete in the reverse complemented *k71*, their fate differed among k71 assemblies containing different complements of mate-pair libraries. Whether increasing the number of mate-pair libraries or swapping out mate-pair libraries with different insert sizes, inconsistent patterns in the completeness of BUSCO genes appeared. These results suggest that different mate-pair library combinations create different scaffolding errors and therefore some BUSCO genes will only be complete with a specific mate-pair or combination of mate-pair libraries. Changes in the BUSCO classification of genes most commonly appeared when mate-pair libraries changed the orientation of the underlying scaffold confirming the effect of mate-pairs on the assembly process and further highlighting the susceptibility of BUSCO classifications to errors due to underlying contig orientation.

#### Conitg-based gene replacement can improve fragmented BUSCO genes in phase I assemblies

We hypothesized that short-read assemblies could be improved by incorporating successful components of different assemblies. Our k-mer-based gene replacement strategy successfully improved 39 of the 79 fragmented BUSCO genes to produce our *cork71* assembly. However, the underlying genomic architecture of the focal genome limits the success of this strategy, as we were unable to fix the 30 additional gene models. While translocating a contig from one assembly to another may fix an assembly error, it also may create additional, new assembly errors highlighting the difficulty of integrating different regions of a genome assembled with different k-mer lengths (whether such an integration is done algorithmically or manually).

### Phase II

#### Erroneous sequence, repeats, and misjoins of contigs can increase duplicated BUSCO genes in hybrid assemblies

We generated hybrid assemblies using quickmerge and compared them to our improved k71 assembly (*cork71*). Our phase II assemblies had higher N50 than *cork71*, however, they also contained a higher number of duplicated BUSCO genes. We found that merging failures between the reference (contigs of the long-read-based *corNpor*) and the query (scaffolds of the short-read-based *cork71*) with same or similar set of BUSCO genes contributed to the inflation of duplicates in our phase II merged assemblies. We observed that setting alignment parameters nonoptimally can halt the merging of a set of phases I and II contigs by reducing or even removing the overlapping portions of an alignment between them.

Large alignment blocks may fail to form if either the reference or query are highly erroneous. We observed that overall number of alignments with a high alignment percentage decreased when the parameter was increased. Moreover, approximately 16% of the nucleotides of the *corNpor* assembly were unconfirmed against Illumina short reads. As contigs of *cork71* (query) are highly accurate at a nucleotide level, the results suggest that contigs of *corNpor* (reference) still possessed sequence errors that favored the formation of many small alignment blocks between the query and the reference. The nonlinear alignment blocks, which we observed when the stringency of alignment length parameter was low, can be explained by genomic repeats because (1) such blocks were filtered out at high stringency and (2) the alignments of small length are more likely to be formed by repeats than due to true homologous regions. Moreover, when merging failure occurs due to any of these conditions, remnants of the unaligned reference sequences can still get dragged into the final merged assembly resulting in additional, duplicated BUSCO genes. This can happen when a single reference sequence overlaps with 2 or more queries at different portions and at least one of the overlaps surpasses the threshold for merging which we observed in our data (SupplementaryFigs.7and9).

We also observed a case in which the erroneous duplication of 23 BUSCO genes occurred when portions of multiple contigs in *corNpor* were present in a single scaffold of *cork71.* And, we found that when the scaffold was manually broken, the duplicated BUSCO genes were converted to single-copy complete genes. These results suggest that the scaffold consisted of misjoined contigs. This also means that the presence of hidden scaffolding error in the short-read-only assembly can also lead to generation of spurious duplicates (SupplementaryFig.10).

All in all, our results have shown that while merging 2 assemblies, optimization of the alignment filtration parameter is vital. Thus, it should be set in a way that minimizes the number of duplicated BUSCO genes in the hybrid assembly. The limitation of this parameter optimization is that it may not improve the number of duplicated genes if these duplicates are due to the presence of hidden scaffolding error from mate-pair libraries used in the original, phase I short-read assembly. In our results, some BUSCO duplicates generated due to mate-pair error persisted in all hybrid assemblies.

We find the pattern of increased duplicated BUSCO genes in phase II assemblies in our study was consistent with the pattern found in the genomes assembled by Xu *et al.* (2021). The authors built a chromosome-level assembly for a diploid, Canadian 2-row malting barley cultivar using Illumina, PacBio, 10X Genomics Chromium linked reads, and Hi-C data following 6 steps. One of the intermediate steps involved the merging of Illumina and PacBio contigs (built with corrected reads and polished with Illumina reads) using quickmerge. In this hybrid assembly, the number of duplicated BUSCO genes (107) was higher than those in genomes of 6-row malting barley cultivar, morex (36) and European 2-row malting barley cultivar, Golden Promise (42) built with Illumina data only.

However, the authors did not interpret their BUSCO scores for any step. We argue that the duplicated BUSCO genes could have increased when generating the phase II assembly due to merging failures since the minimum alignment length was 10 kb, which is potentially high because the long-read contigs were assembled with low coverage data (22X). This coverage is too low to for self-correction (Watson and Warr 2019; Zhang *et al.* 2020) and despite further correcting them with Illumina reads, the contigs will still possess errors (such as insertions and deletions) due to the difficulty in mapping the Illumina reads because of repeats (Watson and Warr 2019) but also due to errors in the underlying contigs. Consequently, not all errors disappear.

Similarly, Das *et al.* (2020) assembled the genome of a diploid snapping turtle, *Chelydra serpentine*. In their study, a phase II assembly was generated by filling gaps in the short-read-only assembly with PacBio long reads (average coverage of 11.4×). This gap-filled assembly was further merged with contigs, independently assembled from Nanopore reads (average coverage of 9.6×), employing quickmerge. The number of duplicated BUSCO genes in *C. serpentine* (70) was higher than in the genomes of related reptiles, including *Chelonia mydas* (21; Illumina-based genome), *Chrysemys picta* (17; Illumina and Sanger-based genome), and *Pelodiscus sinensis* (14; Illumina-based genome), and lower than in *Terrapenemexicana* (253; Illumina and 10X Genomics-based but the protocol is unknown). The “minimum alignment length” of 5 kb was set to merge Illumina scaffolds and Nanopore contigs, which, in our data sets, was large enough to result in merging failures and increased duplicated BUSCO genes. Since mate-pair libraries are also used in their phase I assembly, hidden scaffolding errors could have also contributed to the increased number of duplicated BUSCO genes.

Our results are also useful to interpret an increase in duplicated BUSCO genes found in more complex phase II assemblies generated by the hybridization of assemblies produced by 2 or more assemblers from the same, underlying long-read libraries. For example, Ou *et al.* (2019) generated an assembly of pear tree (“Zhongai 1”) using PacBio CLR reads and an Hi-C library for scaffolding. However, in an intermediate stage, they merged contigs generated by the Canu and WTDBG2 assemblers that were built from the same sequencing libraries. They report that the number of duplicated BUSCO genes from this hybrid assembly was 28% (407) without interpretation. Such a result may indicate that errors in the long-read contigs could have increased the duplicated BUSCO score through merging failure. Based on our results, we argue that such assemblies need to be reanalyzed for their accuracy. Our results suggest that it is useful to keep track of both N50 and BUSCO scores from different stages of the assembly process and interpreting them to evaluate the results of each stage.

#### Underlying scaffolding errors can inflate genome size in phase II assemblies

Our phase II assembly, *filk71*, was created by the hybridization of our phase I, Illumina-based Meraculous assembly with Canu-corrected Nanopore reads, using PBJELLY. This resulted in an increased contig N50 size and drastically lowered the number of assembly gaps. However, the number of duplicated BUSCO genes increased and some genes that were complete in *cork71* became duplicated in *filk71*, which suggests that increase in genome length of *filk71* may be of low fidelity. PBJELLY maps the long reads onto the short-read contigs and fills the gaps in 3 ways. First, a long read may cleanly span a gap within or between scaffolds (SupplementaryFig.11a). Second, a long read extends into a gap without spanning the gap (SupplementaryFig.11b). Third, long reads overfill the gap (SupplementaryFig.11c). In *filk71*, we found numerous cases in which gaps were overfilled. This suggests that scaffolds of Illumina assembly possess hidden scaffolding error. When contigs are misjoined, long reads can align to opposite flanking sequences of a gap between 2 contigs, but those reads cannot align to each other and spuriously expand the genome size.

The problem of overfilling is usually unaccounted by researchers. In the literature, we can find examples that potentially indicate spurious genome size expansion but without any explanation. For example, the gap-filled genome of the snapping turtle assembled by Das *et al.* (2020) had an estimated size of 2.20 Gb. They assembled a phase I genome using Illumina paired-end and mate-pair read libraries with ALLPATHS-LG and subsequently filled the gaps with PBJELLY using error corrected PacBio reads. The size of the genome increased by 186 Mb (from 2.13 to 2.31 Gb), which indicates the gaps are potentially overfilled and this increase in genome size could be a spurious expansion. However, the authors did not quantify the number of overfilled gaps.

All the evidences generated from phase II genome assembly strategies suggest that higher N50 does not necessarily mean higher genome quality, and indicate that BUSCO scores may be informative for genome quality. Researchers typically simply report N50 values and BUSCO scores, without interpretation, and place their analytical emphasis on maximizing N50. Furthermore, they then report high BUSCO “completeness” scores, even if the remaining incomplete BUSCO genes offer a wealth of assembly information that is not being examined or interpreted. A step-wise interpretation of BUSCO scores, along with assembly statistics such as N50 and gap length, can provide researchers with significant information relative to the success of their assembly, and indicate sequencing libraries or analysis algorithms that may be degrading the assembly process. In particular, this type of analysis would make clear when to stop hybridizing different assemblies or assembly components (e.g. specific mate-pair libraries) together.

### Phase III

#### Long-read contig assembly can be tuned for higher contiguity through random sampling of reads

For pure long-read assemblies, we observed that filtering by read length and coverage improves the contiguity of the genome compared to using the maximal number of raw PacBio reads. Generally, researchers use all of the CLR reads that pass a minimum read length threshold for de novo genome assembly. However, CLR reads of extreme length may be of low accuracy due to polymerase errors occurring within the SMRT cell, for example, the polymerase may not loop around the DNA molecule more than once. While the inclusion of reads of extreme length seems desirable for achieving high assembly contiguity, error rate seems to correlate with read length and, consequently, such reads could actually reduce contiguity.

In addition, PacBio reads may be chimeric, i.e. reads from distant parts of the genome joined together. In our analysis, we found a read of long length (>90 kb) that mapped to 2 distinct regions, and the supplementary alignment matched more than 2 kb of the reference with high quality. Excluding these reads is an easy approach to ameliorate this problem. Furthermore, chimeric reads will be rare in the data (Tvedte et al. 2021) and regions of an assembly graph that are linked by such reads will contain low coverage. By randomly sampling all reads down to a base, sufficient level of coverage, these regions of the assembly graph are likely to be excluded, improving the overall assembly. Our result shows that optimizing assembly by subsampling different read sets can help to improve the contiguity of contig-level assemblies. While we provide a program to do the sampling, alternatives, such as seqtk (https://github.com/lh3/seqtk; accessed 2022 Aug 17) are available. Furthermore, tools, such as yacrd (Marijon *et al.* 2020), present an alternative available for reducing chimeric reads in long-read data. Yacrd searches for reads with poor-quality segments based on an all-vs-all alignment of raw reads and selectively filters chimeras. However, it can take a great deal of time and space to process such a set of reads. The subsampling strategy reduces the large data processing time and space consumption for the users. In summary, based on our results, the phase III assembly strategy is the current best state-of-the-art for genome assembly and the resulting contiguity can be tuned by subsampling reads and limiting read lengths.

## Supplementary Material

jkac192_Supplementary_Data

## Data Availability

Raw Illumina and Nanopore reads are available from NCBI under BioProject PRJNA861284. The phases I and II assemblies are hosted on Dryad under DOI 10.5061/dryad.ghx3ffbs3. The custom Python scripts for methods are available in https://bitbucket.org/CatchenLab/scripts_contig_replacement_repo/src/master/. Supplemental material is available at G3 online.
