# Correction to: Evaluating Illumina-, Nanopore-, and PacBio-based genome assembly strategies with the bald notothen, *Trematomus borchgrevinki*

**DOI:** 10.1093/g3journal/jkae214

**Published:** 2024-09-16

This is a correction to: Niraj Rayamajhi, Chi-Hing Christina Cheng, Julian M Catchen, Evaluating Illumina-, Nanopore-, and PacBio-based genome assembly strategies with the bald notothen, *Trematomus borchgrevinki*, *G3 Genes|Genomes|Genetics*, Volume 12, Issue 11, November 2022, jkac192, https://doi.org/10.1093/g3journal/jkac192.

Data was split into two NCBI BioProjects, and the IDs are provided here as an update to the BioProject listed in the Data Availability Statement.

Raw Nanopore long-read, and Illumina short-read data for the male *T. borchgrevinki* assemblies are at https://www.ncbi.nlm.nih.gov/bioproject/PRJNA861284.

Raw PacBio Sequel II long-read data for the female *T. borchgrevinki* assemblies are available at https://www.ncbi.nlm.nih.gov/bioproject/PRJNA907802.

These details have been corrected only in this correction notice to preserve the published version of record.

