# Supplementary material for: Evaluating Illumina-, Nanopore-, and PacBio-based genome assembly strategies with the bald notothen, Trematomus borchgrevinki
Source: G3 (Bethesda). 2022 Jul 29;12(11):jkac192. doi: 10.1093/g3journal/jkac192 (PMC9635638; doi:10.1093/g3journal/jkac192)
Supplement: jkac192_Supplementary_Data [file jkac192_supplementary_data.pdf]

Supplementary Figures

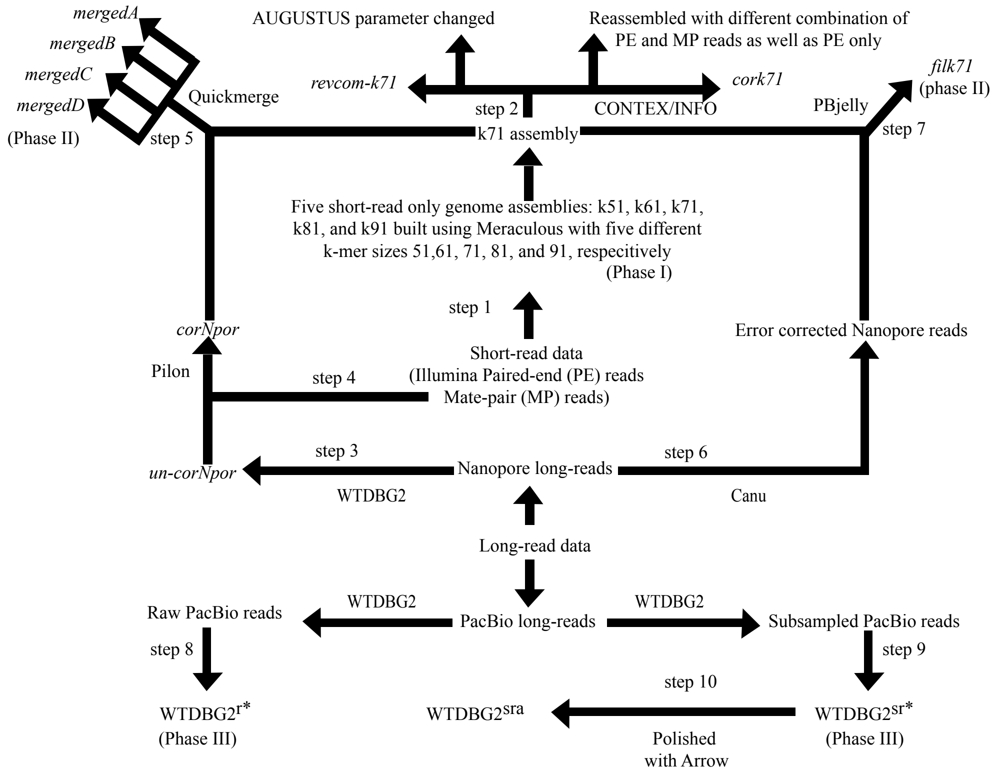

Figure S1. Flow chart showing ten steps employed to assemble *de novo* genomes with phase I, II, and III strategies by using Illumina short-reads, Oxford Nanopore long-reads, and Pacific Biosciences long-reads. Step 1: Five short-read assemblies were built with different k-mer sizes of 51 to 91bp using paired-end and mate-pair short-reads. Step 2a: for the k71 assembly, scaffolds were reverse complemented (*revcom-k71*); Step 2b: BUSCO analysis was performed while changing the AUGUSTUS parameter; Step 2c: fragmented BUSCO genes replaced with their complete version using CONTEX/INFO scripts; Stedp 2d: reassemblies were completed with different combinations of mate-pair and paired-end data. Step 3: Nanopore long-reads were assembled with WTDBG2 to produce low coverage, contig-level assembly (*un-corNpor*). Step 4: The *un-corNpor* was polished with short-reads using Pilon to create an error-corrected assembly (*corNpor*). Step 5: The k71 and *corNpor* assemblies were merged as *query* and *reference*, respectively, using Quickmerge by changing the minimum length of alignment in 4 different ways (0, 1000, 5000, 10000) at a minimum alignment identity of 95% to produce hybrid assemblies *mergedA, B, C,* and *D*. Step 6: Nanopore long-reads were corrected with Canu. Step 7: Gaps were filled using the error-corrected Nanopore reads with PBjelly. Step 8: Raw PacBio long-reads were assembled natively using WTDBG2^{r*}. Step 9: Raw PacBio reads were subsampled and assembled to generate contig-level assembly, WTDBG2^{sr*}. Step 10: Error correction was performed on the assembly from step 9 to generate a polished assembly, WTDBG2^{sra}.

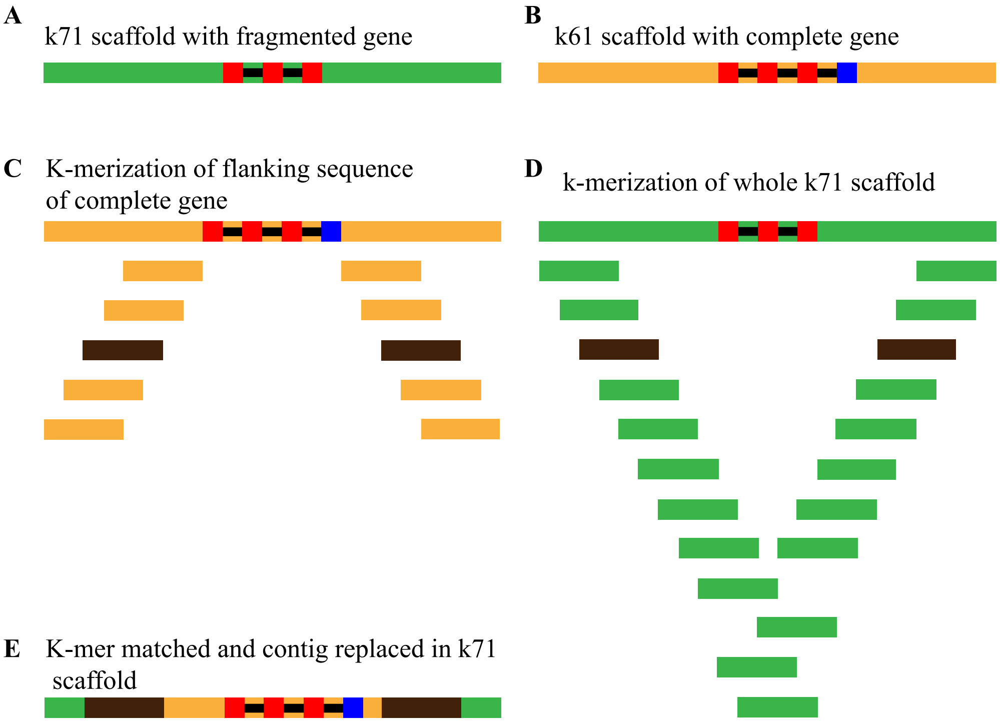

Figure S2. The five core steps of the CONTEX algorithm. A) Identify the k71 scaffold that contains a fragmented BUSCO gene. B) Identify a scaffold in an alternative assembly (e.g., k61) containing a complete version of the same BUSCO gene. C) K-merize the flanking sequences of the complete BUSCO gene. D) K-merize the whole k71 scaffold and search for matching k-mers in the alternative flanking sequence. E) If the k-mers match, replace the contig within the k71 scaffold with the contig from the alternative assembly.

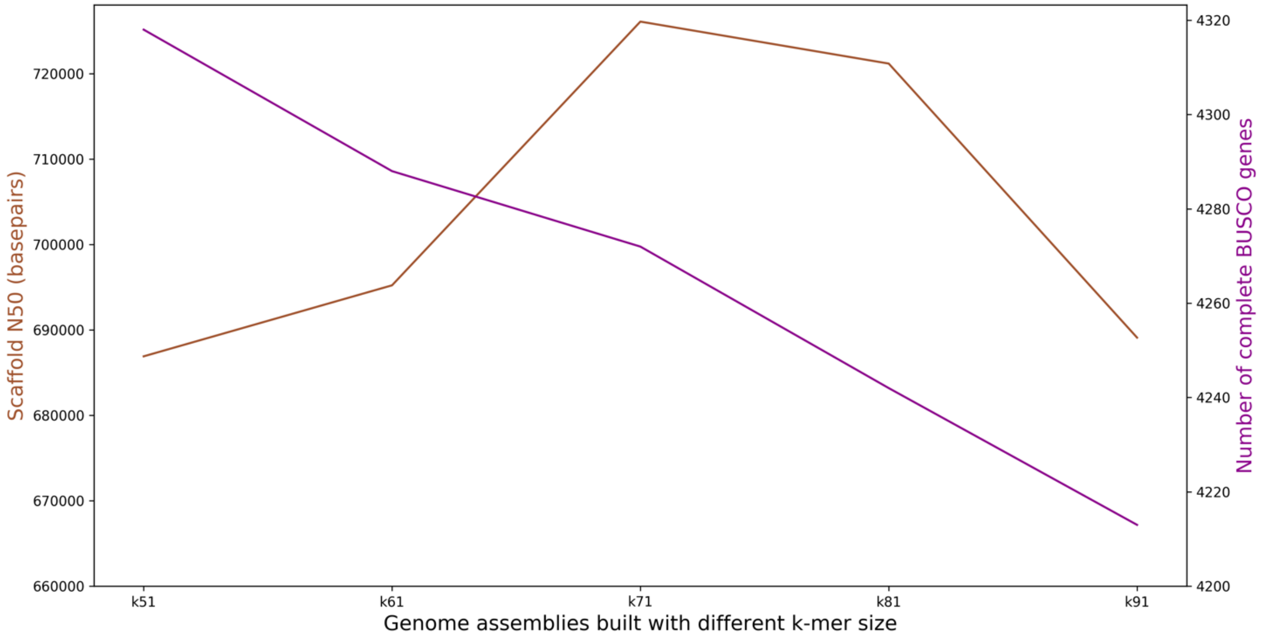

Figure S3. Assembly with high contiguity showed low BUSCO gene completeness. This figure shows the contiguity and the completeness of BUSCO genes (specific to Actinopterygii clade), for the short-read only assemblies of *Trematomus borchgrevinki* built with five different k-mer sizes ranging from 51 to 91.

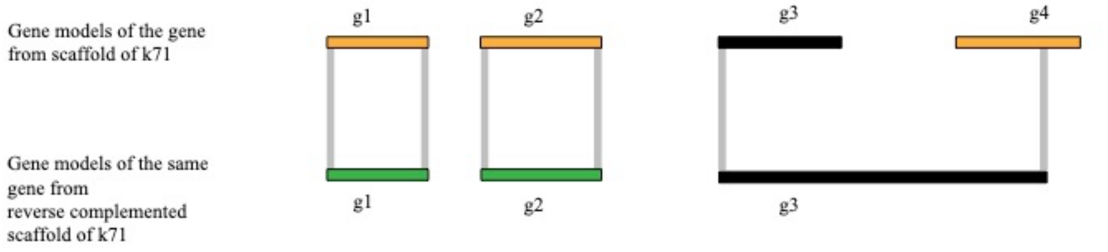

Figure S4. Reverse complementing a scaffold reduced the number of gene models and increased the length of one of those gene models (g3, black color). Genes g1-g4 are transcripts (gene models) of the same underlying BUSCO gene in k71. After reverse complementing the scaffold containing these gene models, g3 and g4 are merged, resulting in a longer version of g3.

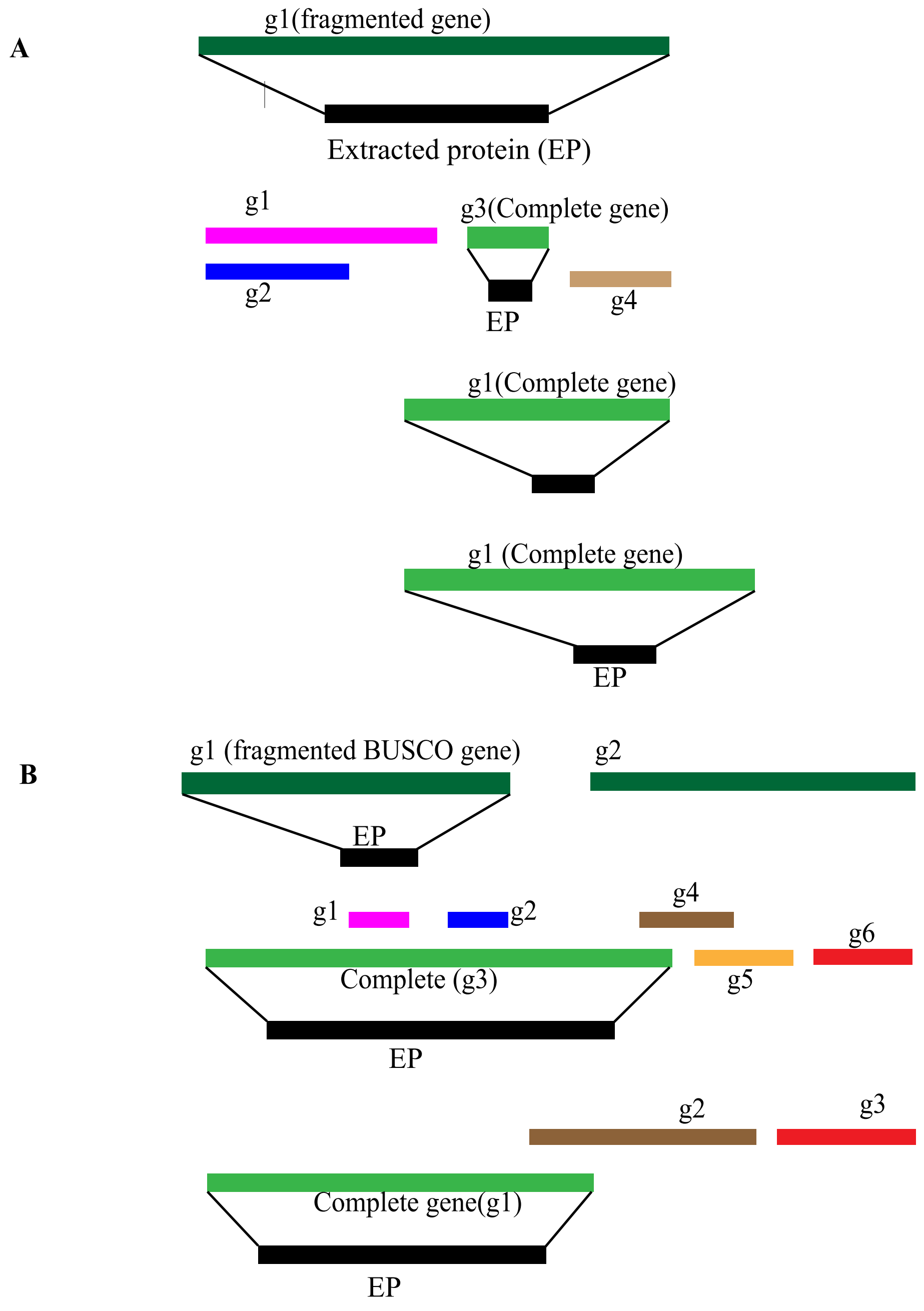

Figure S5. Change in gene length with `--singlestrand=true` parameter in AUGUSTUS. Dark green gene models are predicted by AUGUSTUS with `--singlestrand=false` whereas green models and all other colors are predicted by AUGUSTUS with `--singlestrand=true`. A) The fragmented gene model (g1) became complete (light green g3 and g1) through a reduction of gene size. The gene coordinates of the complete versions fell within those of the fragmented version or one of its coordinates shifted outside the boundary of fragmented version. B) The fragmented gene (g1) became complete (light green g1) through an increase in size when the parameter was true. The complete versions overlapped other gene models of the same gene.

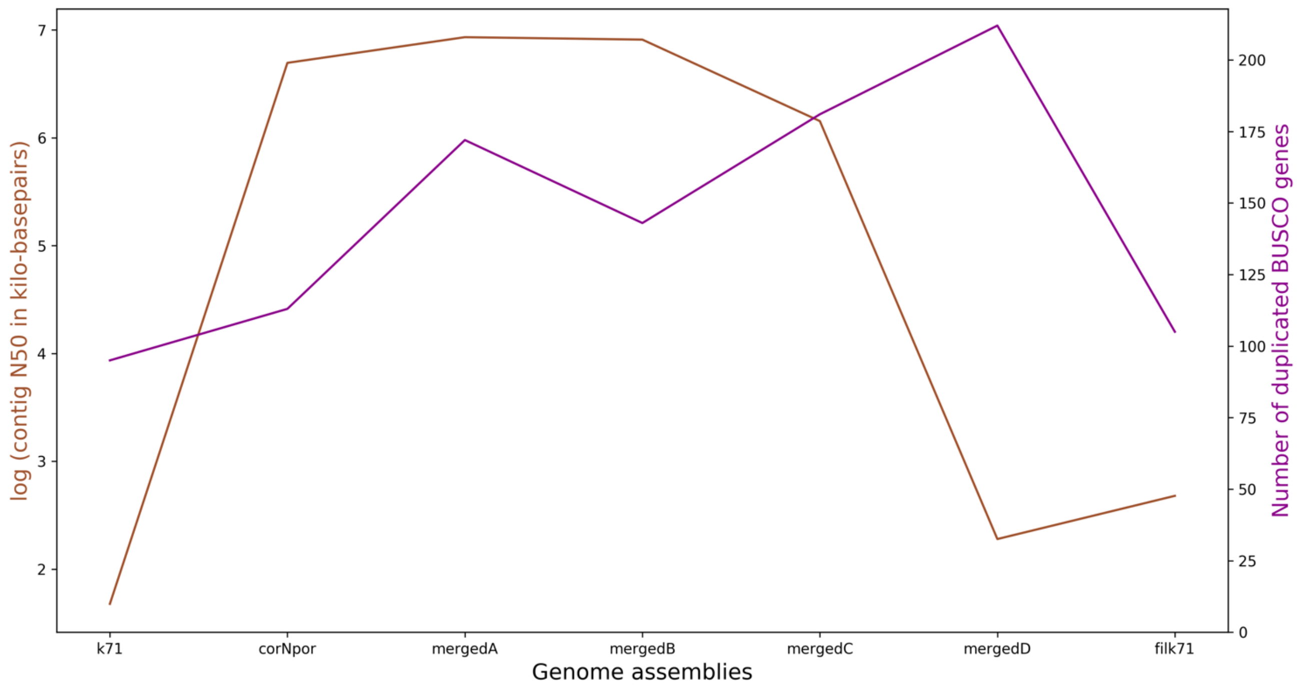

Figure S6. The number of duplicated BUSCO genes and contig N50 increased in Quickmerge-based hybrid assemblies (mergedA, B, C, and D) compared to their query (k71) and reference (corNpor) assemblies as well as in gap-filled, PBjelly-based hybrid assembly (filk71) compared to k71 assembly with unfilled gaps.

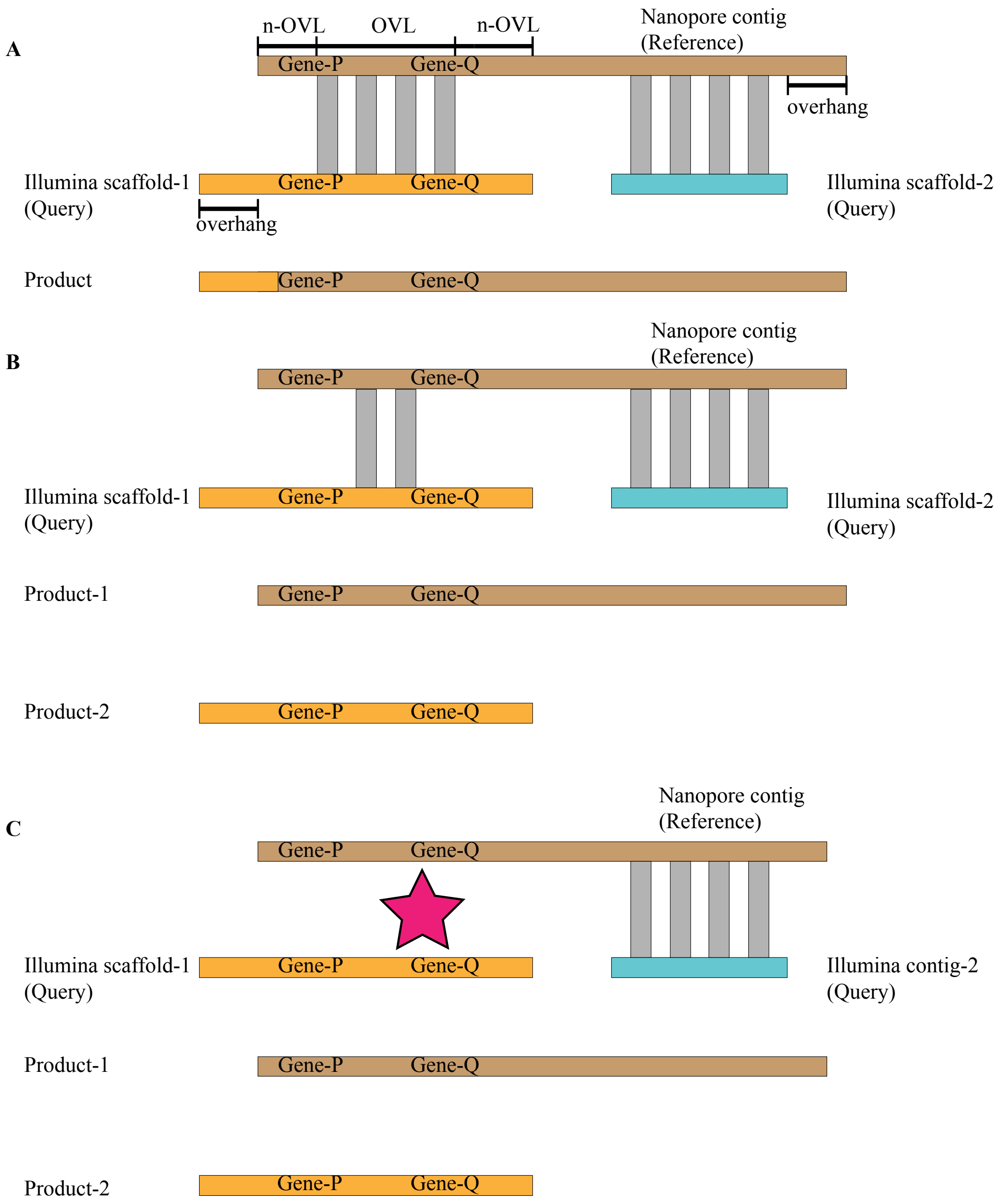

Figure S7. Duplication of BUSCO genes via the Quickmerge algorithm. **A**) Successful alignment and merging of one nanopore and two Illumina scaffolds without generating duplicated BUSCO genes. When a query contig (e.g., Illumina scaffold-1) is aligned to a reference contig (Nanopore contig), the alignment has three components: overhang, overlapped but unaligned (n-OVL), and overlapped and aligned (OVL) sequence (Vertical grey bars represent aligned regions). Quickmerge uses the ratio of OVL/n-OVL to determine if the query and reference contigs should be merged (product). Note if overhangs are present in the reference and/or the query, they are retained in the product. **B**) One nanopore contig aligned to two Illumina scaffolds in which Illumina scaffold-2 merged with the nanopore contig; however, Illumina scaffold-1 failed to merge. Consequently, two products were produced with the same BUSCO genes. **C**) Alignment between Illumina scaffold-1 failed with nanopore contig. Consequently, two products are produced with the same BUSCO gene set. Star sign indicates alignment failure.

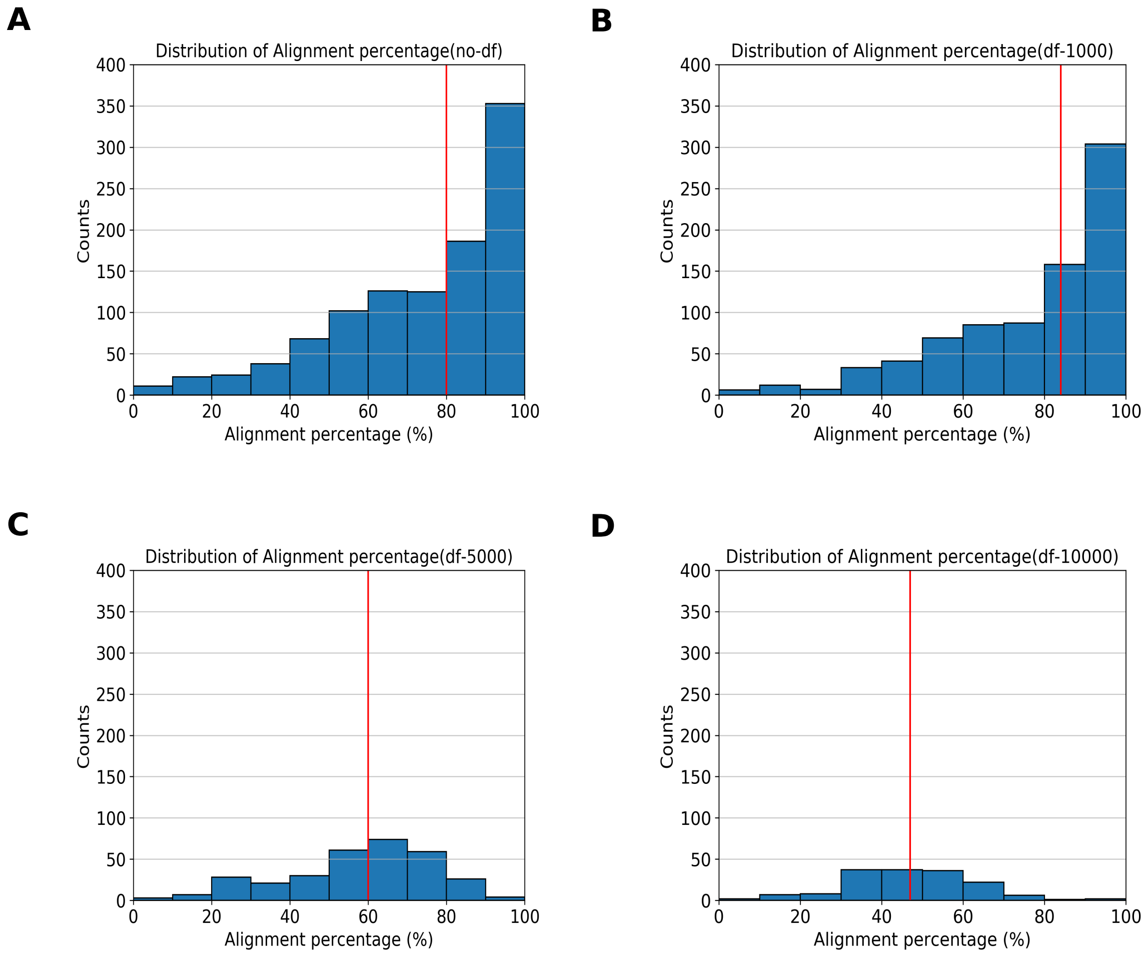

Figure S8. Distribution of alignments generated with different parameter settings when merging assemblies to create a phase II hybrid assembly. The plot shows the distribution of alignment percentage for different minimum length of alignment (L) parameters employed in the Mummer delta-filter (df) program when setting a 95% minimum alignment identity. For A) L equals to 0, B) L equals to 1000, C) L = 5000, and D) L equals to 10000. X-axis represents alignment percentage whereas y-axis represents counts of those alignment percentages. Red line represents median of the alignment percentage. The number of high percentage alignments decreased with an increase in the stringency of minimum length of the alignment (shown in figure A to D).

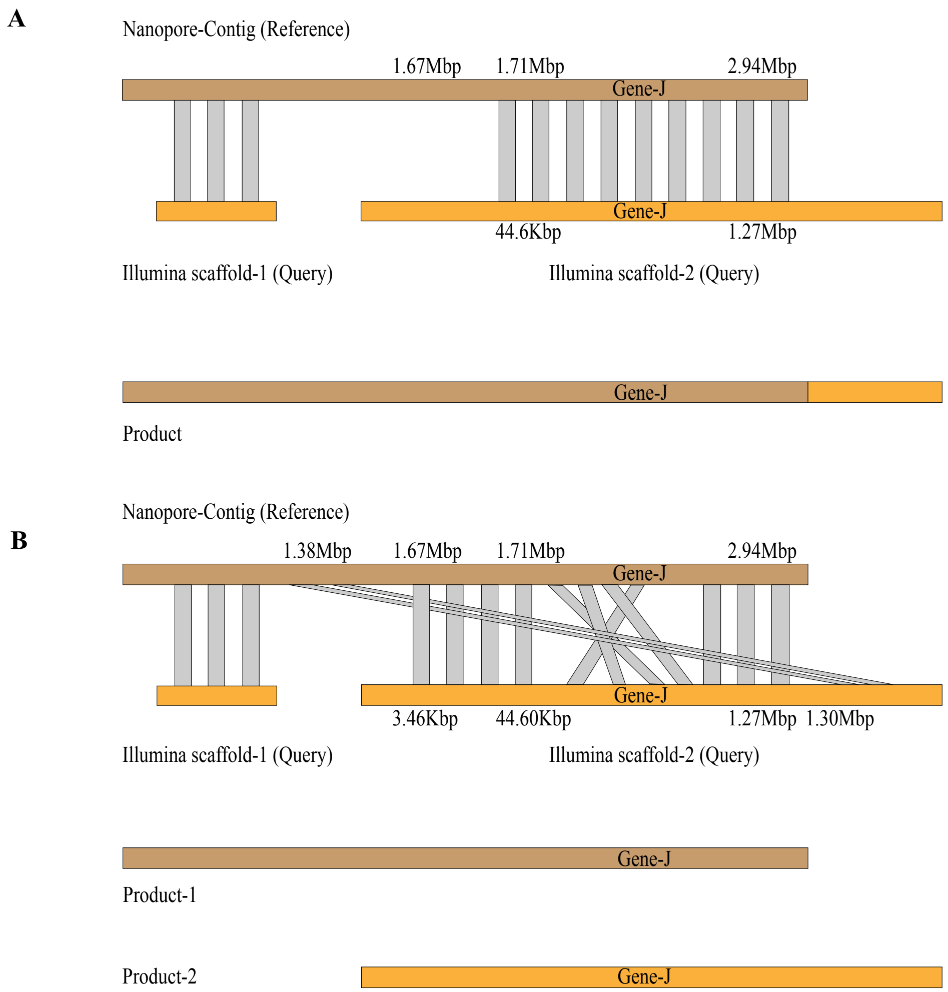

Figure S9. The disruption of the linear order of nucmer alignments between the query (Illumina scaffold) and reference (Nanopore) contigs, as implemented by Quickmerge (using the set of Mummer alignment tools), resulting in duplicated BUSCO gene (e.g. Gene-J). A) Successful, linearly ordered alignment and merged product. B) The disrupted order of alignments between query and reference contigs due to small and spurious alignments leading to merging failure and a duplicated BUSCO gene.

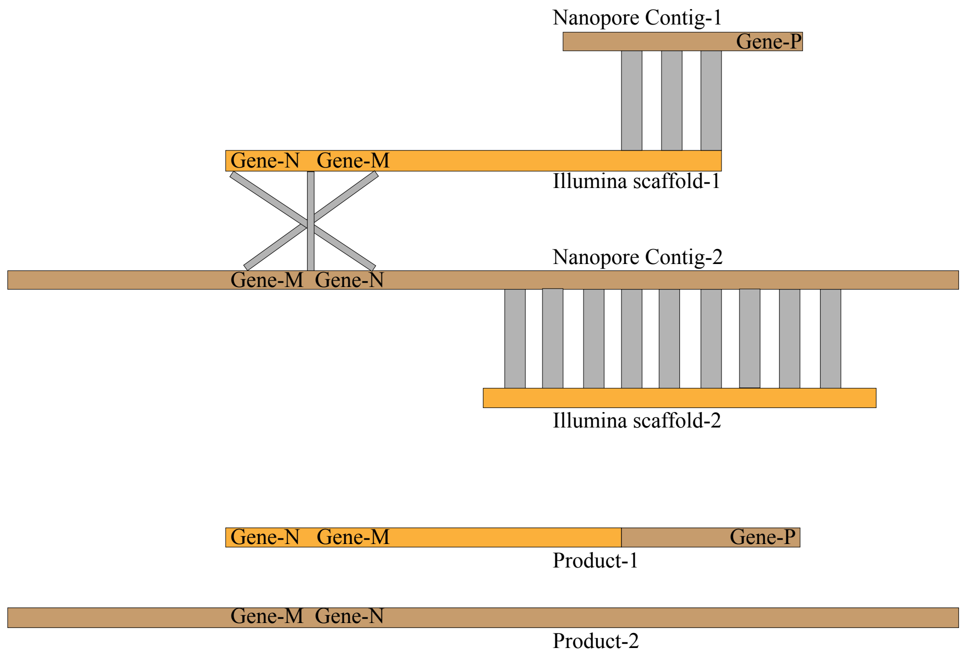

Figure S10. Duplication of BUSCO genes (e.g. Gene-M, Gene-N) in the merged, phase II assembly due to the effect of mis-joined contigs in Illumina scaffold-1 (composed of portions of different Nanopore contigs). A) Illumina scaffold-1 composed of distantly related contigs aligned and merged to at-least two Nanopore contigs-1 and -2. B) The merged assembly contained duplicated BUSCO genes.

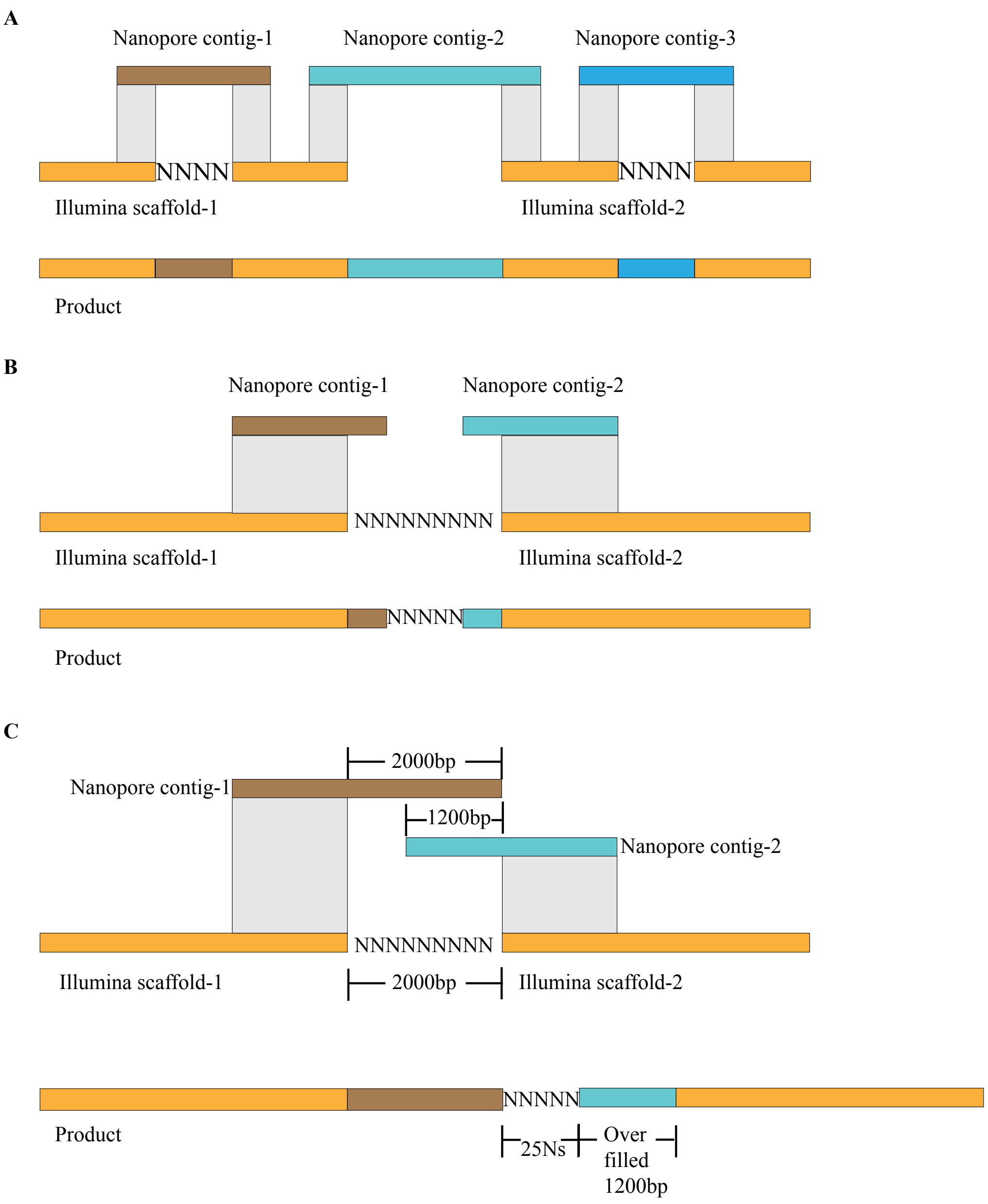

Figure S11. The gap-filling process of PBJELLY. A) When Nanopore long-reads span across gaps (Ns), the gap is filled. B) When a long-read extends into the gap, the gap is partially filled. C) When distinct nanopore long-reads extend into the gap from either side, but do not align with one another, the gap is extended according to the lengths of the individual reads, potentially overfilling the gap (and an additional gap of 25 Ns is added by PBJELLY). For example, if the total length of a gap is 2000bp prior to merging, and nanopore reads extend into the gap 2000bp on one side and 1200bp on the other, then the total gap is extended to 3200bp (plus 25bp of Ns).

A

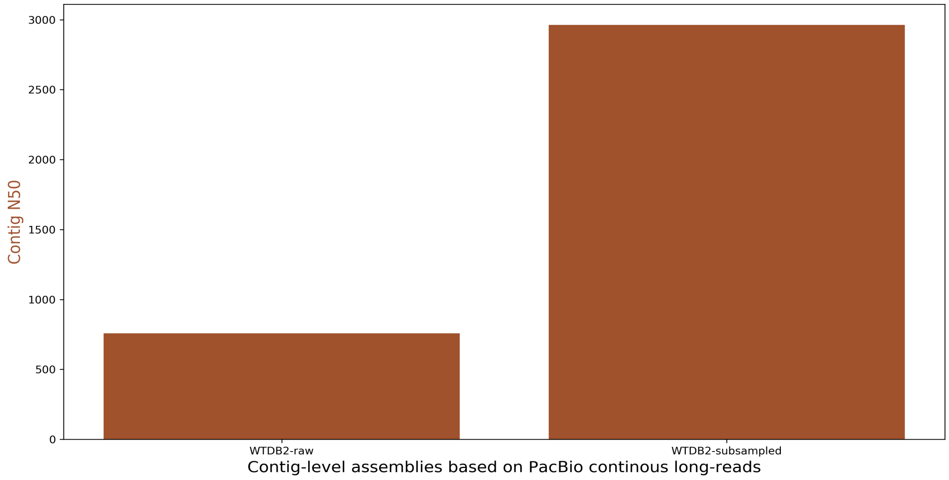

B

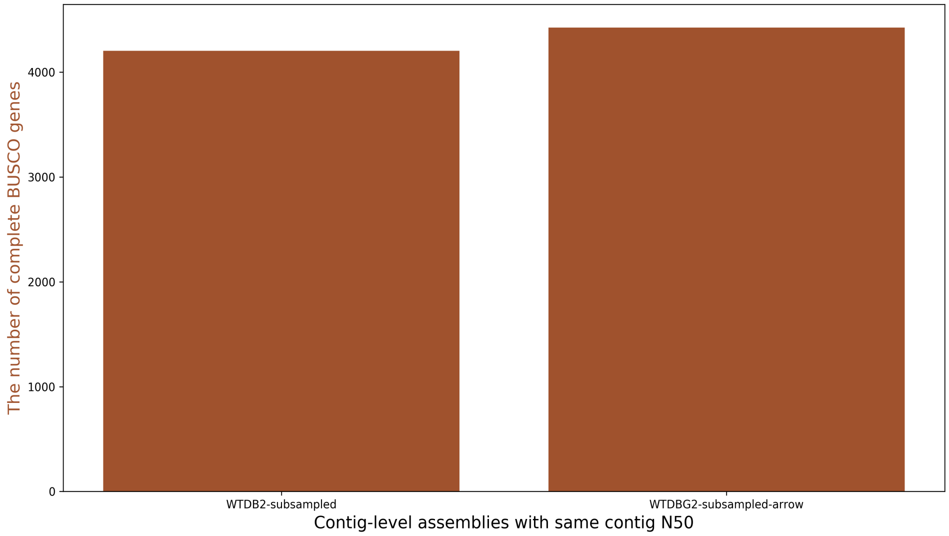

Figure S12. Subsampling of PacBio contiguous long-reads can increase contiguity for contig-level assembly and such assemblies' BUSCO gene completeness can be increased by polishing through self-error correction protocol. A) This figure shows that assembly WTDBG2-subsampled assembly (WTDBG2^{Sr*} in Table 1) built by subsampling PacBio reads has high contiguity metric N50 than WTDBG2-raw (WTDBG2^{r*} in Table 1) built with raw reads. B) This figure shows that error corrected, subsampled assembly WTDBG2-subsampled-arrow (WTDBG2^{Sra} in Table 1) has more BUSCO gene completeness than uncorrected, WTDBG2-subsampled.

Supplementary Tables

Table S1. Genome statistics for five different short-read-only genome assemblies built with five different k-mer sizes ranging from 51 to 91.

Assembly	K-mer size	Number of scaffolds	Scaffold N50	Scaffold L50	Total scaffold length	Max. scaffold length	GC %	N's per 100 Kbp	Contig N50	Contig L50	Total contig length
k51	51	8,018	686,912	294	744,619,395	4,513,143	40.12	25,658.16	5,102	28,468	553,563,782
k61	61	8,561	695,226	285	744,343,530	5,414,719	40.13	24,808.03	5,323	27,463	559,686,532
k71	71	9,399	726,105	271	746,021,077	4,901,101	40.16	23,813.61	5,374	27,669	568,366,538
k81	81	10,579	721,191	257	745,435,592	5,652,300	40.19	22,871.49	5,404	27,739	574,943.361
k91	91	13,160	689,102	272	741,361,822	5,102,311	40.2	22,243.97	5,183	28,922	576,453,487

Table S2. Summary of Benchmarking Universal Single-Copy Orthologs (BUSCOs) specific to Actinopterygii clade in the five different short-read-only genome assemblies built with five different k-mer sizes ranging from 51 to 91.

Assembly	K-mer size	Complete	Complete and single-copy	Complete and duplicated	Fragmented	Missing	Total BUSCO groups searched
k51	51	4318 (94.2%)	4221 (92.1%)	97 (2.1%)	94 (2.1%)	172 (3.8%)	4584
k61	61	4288 (93.5%)	4186 (91.3%)	102 (2.2%)	118 (2.6%)	178 (3.9%)	4584
k71	71	4272 (93.2%)	4177 (91.1%)	95 (2.1%)	130 (2.8%)	182 (4.0%)	4584
k81	81	4242 (92.5%)	4146 (90.4%)	96 (2.1%)	150 (3.3%)	192 (4.2%)	4584
k91	91	4213 (92.0%)	4110 (89.7%)	103 (2.2%)	148 (3.2%)	223 (4.9%)	4584

Table S3. The number of BUSCO genes in k71 that converted the status from one version of the gene to another in reverse complemented k71 (revcomp-k71).

	Complete in revcomp-k71	Duplicated in revcomp-k71	Fragmented in revcomp-k71	Missing in revcomp-k71
Complete in k71	4111	19	20	27
Duplicated in k71	19	76	0	0
Fragmented in k71	29	0	98	3
Missing in k71	12	0	3	167

Table S4. The status of twenty-nine fragmented BUSCO genes from k71 in reverse complemented k71 (revcomp-k71).

**Gene**	**revcomp-k71**
EOG090C031F	Complete
EOG090C06A3	Complete
EOG090C09GB	Complete
EOG090C0BB3	Complete
EOG090C0CPN	Complete
EOG090C0CYM	Complete
EOG090C0E4A	Complete
EOG090C0FHB	Complete
EOG090C0FKI	Complete
EOG090C0GDD	Complete
EOG090C01H0	Complete
EOG090C04AG	Complete
EOG090C04VT	Complete
EOG090C08YF	Complete
EOG090C0AN8	Complete
EOG090C0B2Q	Complete
EOG090C0DUI	Complete
EOG090C0FHE	Complete
EOG090C03HW	Complete
EOG090C04JV	Complete
EOG090C05LL	Complete
EOG090C0FY1	Complete
EOG090C03FY	Complete
EOG090C0ARU	Complete
EOG090C0E9K	Complete
EOG090C01VQ	Complete
EOG090C03H9	Complete
EOG090C07FU	Complete
EOG090C0AHG	Complete

Table S5. Genome statistics for six different reassembled genomes built with k-mer size of k71.

Reassembled k71	Nuber of Scaffolds	Scaffold N50	Total scaffold length	N's per 100kbp	Number of contigs	Contig N50	Total contig length
PE	92,837	11,287	587,324,760	4,792.79	104,967	7,668	507,922,453
PE+5Kbp	22,019	133,778	696,585,992	18,074.86	108,006	7,240	506,616,768
PE+7Kbp	23,130	164,338	736,792,643	23,032.04	108,599	7,304	504,701,234
PE+12Kbp	31,969	152,151	775,606,865	27,490.87	111,141	7,078	502,856,587
PE+5+7Kbp	12,786	375,608	723,717,036	21,332.22	112,871	6,741	502,880,470
PE+5+7+12Kbp	9,397	718,560	745,779,012	23,786.12	116,549	6,381	500,201,938

PE indicates k71 reassembled with paired-end data only
PE+5Kbp indicates k71 reassembled with paired-end data plus mate-pair reads with 5Kbp insert size
PE+7Kbp indicates k71 reassembled with paired-end data plus mate-pair reads with 7Kbp insert size
PE+12Kbp indicates k71 reassembled with paired-end data plus mate-pair reads with 12Kbp insert size
PE+5+7Kbp indicates k71 reassembled with paired-end data plus mate-pair reads with 5Kp and 7Kbp insert sizes
PE+5+7+12Kbp indicates k71 reassembled with paired-end data plus mate-pair reads with 5Kp, 7Kbp, and 12Kbp insert sizes

Table S6. Summary of Benchmarking Universal Single-Copy Orthologs (BUSCOs) specific to Actinopterygii clade in the six different reassembled genomes built with k-mer size of k71.

Reassembled k71	Complete	Complete and single-copy	Complete and duplicated	Fragmented	Missing	Total BUSCO groups searched
PE	2918 (63.7%)	2860 (62.4%)	58 (1.3%)	962 (21.0%)	704 (15.3%)	4584
PE+5Kbp	4093 (89.3%)	4001 (87.3%)	92 (2.0%)	278 (6.1%)	213 (4.6%)	4584
PE+7Kbp	4149 (90.5%)	4058 (88.5%)	91 (2.0%)	210 (4.6%)	225 (4.9%)	4584
PE+12Kbp	4051 (88.4%)	3957 (86.3%)	94 (2.1%)	261 (5.7%)	272 (5.9%)	4584
PE+5+7Kbp	4257 (92.8%)	4169 (90.9%)	88 (1.9%)	136 (3.0%)	191 (4.2%)	4584
PE+5+7+12Kbp	4267 (93.1%)	4182 (91.2%)	85 (1.9%)	129 (2.8%)	188 (4.1%)	4584

Table S7. The status of twenty-nine BUSCO genes (fragmented in k71 but complete in reverse complemented k71) across six different k71 reassembled genomes

Gene	PE	PE+5kbp	PE+7Kbp	PE+12Kbp	PE+5+7Kbp	PE+5+7+12Kbp
EOG090C031F	Complete	Complete	Complete	Complete	Complete	Complete
EOG090C06A3	Complete	Complete	Complete	Complete	Fragmented	Fragmented
EOG090C09GB	Complete	Fragmented	Complete	Fragmented	Complete	Complete
EOG090C0BB3	Complete	Fragmented	Fragmented	Fragmented	Complete	Fragmented
EOG090C0CPN	Complete	Fragmented	Fragmented	Complete	Fragmented	Fragmented
EOG090C0CYM	Complete	Complete	Complete	Complete	Complete	Fragmented
EOG090C0E4A	Complete	Complete	Fragmented	Fragmented	Complete	Fragmented
EOG090C0FHB	Complete	Fragmented	Fragmented	Complete	Complete	Fragmented
EOG090C0FKI	Complete	Complete	Complete	Fragmented	Complete	Fragmented
EOG090C0GDD	Complete	Complete	Complete	Complete	Complete	Fragmented
EOG090C01H0	Fragmented	Complete	Complete	Complete	Complete	Complete
EOG090C04AG	Missing	Complete	Fragmented	Complete	Fragmented	Complete
EOG090C04VT	Fragmented	Complete	Fragmented	Fragmented	Complete	Fragmented
EOG090C08YF	Fragmented	Complete	Fragmented	Complete	Complete	Complete
EOG090C0AN8	Missing	Complete	Complete	Missing	Complete	Complete
EOG090C0B2Q	Fragmented	Complete	Fragmented	Complete	Complete	Complete
EOG090C0DUI	Fragmented	Complete	Complete	Complete	Fragmented	Complete
EOG090C0FHE	Fragmented	Complete	Fragmented	Complete	Fragmented	Complete
EOG090C03HW	Fragmented	Missing	Complete	Fragmented	Fragmented	Fragmented
EOG090C04JV	Fragmented	Fragmented	Complete	Complete	Complete	Fragmented
EOG090C05LL	Fragmented	Fragmented	Complete	Fragmented	Complete	Fragmented
EOG090C0FY1	Fragmented	Fragmented	Complete	Complete	Fragmented	Complete
EOG090C03FY	Fragmented	Fragmented	Fragmented	Complete	Complete	Complete
EOG090C0ARU	Fragmented	Fragmented	Fragmented	Complete	Complete	Fragmented
EOG090C0E9K	Fragmented	Fragmented	Fragmented	Fragmented	Fragmented	Complete
EOG090C01VQ	Missing	Fragmented	Fragmented	Missing	Fragmented	Fragmented
EOG090C03H9	Missing	Fragmented	Missing	Missing	Fragmented	Fragmented
EOG090C07FU	Missing	Fragmented	Fragmented	Missing	Fragmented	Complete
EOG090C0AHG	Fragmented	Fragmented	Fragmented	Fragmented	Fragmented	Fragmented

PE indicates k71 reassembled with paired-end data only
PE+5Kbp indicates k71 reassembled with paired-end data plus mate-pair reads with 5Kbp insert size
PE+7Kbp indicates k71 reassembled with paired-end data plus mate-pair reads with 7Kbp insert size
PE+12Kbp indicates k71 reassembled with paired-end data plus mate-pair reads with 12Kbp insert size
PE+5+7Kbp indicates k71 reassembled with paired-end data plus mate-pair reads with 5Kp and 7Kbp insert sizes
PE+5+7+12Kbp indicates k71 reassembled with paired-end data plus mate-pair reads with 5Kp, 7Kbp, and 12Kbp insert sizes

Table S8. The number of complete versions for twenty-nine BUSCO genes (fragmented in k71 but complete in reverse complemented k71) across six different k71 reassembled genomes

revcomp-k71	PE	PE+5Kbp	PE+7Kbp	PE+12Kbp	PE+5+7Kbp	PE+5+7+12Kbp
29/29	10/29	14/29	13/29	16/29	17/29	13/29

Table S9. The status of twenty-nine BUSCO genes (fragmented in k71 but complete in reverse complemented k71) across six different k71 reassembled genomes and their reverse complemented versions

Gene	PE+5Kbp	revcomp-PE+5Kbp	PE+7Kbp	revcomp-PE+7Kbp	PE+12Kbp	revcomp-PE+12Kbp	PE +5+7Kbp	revcomp-PE+5+7Kp	PE +5+7+12Kbp
EOG090C031F	Complete	Complete	Complete	Fragmented	Complete	Fragmented	Complete	Complete	Complete
EOG090C06A3	Complete	Complete	Complete	Complete	Complete	Complete	Fragmented	Complete	Fragmented
EOG090C09GB	Fragmented	Complete	Complete	Complete	Fragmented	Complete	Complete	Complete	Complete
EOG090C0BB3	Fragmented	Complete	Fragmented	Complete	Fragmented	Complete	Complete	Fragmented	Fragmented
EOG090C0CPN	Fragmented	Fragmeted	Fragmented	Complete	Complete	Fragmented	Fragmented	Complete	Fragmented
EOG090C0CYM	Complete	Complete	Complete	Complete	Complete	Complete	Complete	Fragmented	Fragmented
EOG090C0E4A	Complete	Fragmeted	Fragmented	Complete	Fragmented	Complete	Complete	Complete	Fragmented
EOG090C0FHB	Fragmented	Complete	Fragmented	Complete	Complete	Fragmented	Complete	Fragmented	Fragmented
EOG090C0FKI	Complete	Complete	Complete	Complete	Fragmented	Complete	Complete	Complete	Fragmented
EOG090C0GDD	Complete	Complete	Complete	Complete	Complete	Complete	Complete	Fragmented	Fragmented
EOG090C01H0	Complete	Fragmeted	Complete	Fragmented	Complete	Fragmented	Complete	Complete	Complete
EOG090C04AG	Complete	Complete	Fragmented	Complete	Complete	Fragmented	Fragmented	Fragmented	Complete
EOG090C04VT	Complete	Fragmeted	Fragmented	Complete	Fragmented	Complete	Complete	Fragmented	Fragmented
EOG090C08YF	Complete	Complete	Fragmented	Complete	Complete	Fragmented	Complete	Complete	Complete
EOG090C0AN8	Complete	Complete	Complete	Fragmented	Missing	Missing	Complete	Complete	Complete
EOG090C0B2Q	Complete	Fragmeted	Fragmented	Complete	Complete	Fragmented	Complete	Complete	Complete
EOG090C0DUI	Complete	Fragmeted	Complete	Fragmented	Complete	Missing	Fragmented	Fragmented	Complete
EOG090C0FHE	Complete	Fragmeted	Fragmented	Complete	Complete	Fragmented	Fragmented	Complete	Complete
EOG090C03HW	Missing	Fragmeted	Complete	Complete	Fragmented	Fragmented	Fragmented	Complete	Fragmented
EOG090C04JV	Fragmented	Complete	Complete	Complete	Complete	Fragmented	Complete	Fragmented	Fragmented
EOG090C05LL	Fragmented	Fragmeted	Complete	Fragmented	Fragmented	Complete	Complete	Fragmented	Fragmented
EOG090C0FY1	Fragmented	Complete	Complete	Fragmented	Complete	Fragmented	Fragmented	Complete	Complete
EOG090C03FY	Fragmented	Complete	Fragmented	Complete	Complete	Fragmented	Complete	Fragmented	Complete
EOG090C0ARU	Fragmented	Complete	Fragmented	Complete	Complete	Fragmented	Complete	Complete	Fragmented
EOG090C0E9K	Fragmented	Fragmeted	Fragmented	Complete	Fragmented	Complete	Fragmented	Fragmented	Complete
EOG090C01VQ	Fragmented	Fragmeted	Fragmented	Fragmented	Missing	Missing	Fragmented	Fragmented	Fragmented

EOG090C03H9	Fragmented	Fragmeted	Missing	Missing	Missing	Missing	Fragmented	Complete	Fragmented
EOG090C07FU	Fragmented	Fragmeted	Fragmented	Fragmented	Missing	Fragmented	Fragmented	Fragmented	Complete
EOG090C0AHG	Fragmented	Fragmeted	Fragmented	Fragmented	Fragmented	Fragmented	Fragmented	Complete	Fragmented

PE indicates k71 reassembled with paired-end data only
PE+5Kbp indicates k71 reassembled with paired-end data plus mate-pair reads with 5Kbp insert size
revcomp- PE+5Kbp indicates reverse complemented PE+5Kbp
PE+7Kbp indicates k71 reassembled with paired-end data plus mate-pair reads with 7Kbp insert size
revcomp- PE+7Kbp indicates reverse complemented PE+7Kbp
PE+12Kbp indicates k71 reassembled with paired-end data plus mate-pair reads with 12Kbp insert size
revcomp- PE+12Kbp indicates reverse complemented PE+12Kbp
PE+5+7Kbp indicates k71 reassembled with paired-end data plus mate-pair reads with 5Kp and 7Kbp insert sizes
revcomp- PE+5+7Kbp indicates reverse complemented PE+5+7Kbp
PE+5+7+12Kbp indicates k71 reassembled with paired-end data plus mate-pair reads with 5Kp, 7Kbp, and 12Kbp insert sizes

Table S10. Thirty-nine BUSCO genes fixed (i.e. convert from fragmented to complete versions) using CONTEX

**Fragmented BUSCO gene in k71**	**Source assembly used to fix the fragmented BUSCO gene**	**Status (after editing)**
EOG090C00H3	K51	Complete
EOG090C01CE	K51	Complete
EOG090C01JC	K51	Complete
EOG090C01QA	K51	Complete
EOG090C01QT	K51	Complete
EOG090C01T5	K51	Complete
EOG090C01T6	K51	Complete
EOG090C02EI	K51	Complete
EOG090C02LX	K51	Complete
EOG090C02NA	K51	Complete
EOG090C02NK	K51	Complete
EOG090C02ZZ	K51	Complete
EOG090C03AV	K51	Complete
EOG090C03P2	K51	Complete
EOG090C03TB	K51	Complete
EOG090C04IH	K51	Complete
EOG090C04LE	K51	Complete
EOG090C04O0	K51	Complete
EOG090C04U0	K51	Complete
EOG090C0502	K51	Complete
EOG090C0563	K61	Complete
EOG090C05AY	K51	Complete
EOG090C05M1	K51	Complete
EOG090C06C9	K51	Complete
EOG090C06X2	K51	Complete
EOG090C0879	K51	Complete
EOG090C09IE	K51	Complete
EOG090C09LR	K51	Complete
EOG090C09XA	K51	Complete
EOG090C0AEQ	K51	Complete
EOG090C0AX8	K51	Complete
EOG090C0BAB	K51	Complete
EOG090C0CJD	K51	Complete

EOG090C0DGW	K51	Complete
EOG090C0A7K	K61	Complete
EOG090C0BZH	K61	Complete
EOG090C04CH	K81	Complete
EOG090C02PR	K91	Complete
EOG090C0C11	K91	Complete

Table S11. Genome statistics for assemblies built with raw PacBio data as well as subsampled data

Data	Data amount (Gbp)	Min (Kbp)	Max (Kbp)	N50 (Mb)	# contigs	largest contig (Mb)	Total length (Mb)	L50	Estimated genome size-s	Estimated genome size-a
Raw	181.4	N/A	N/A	0.76	10848	13. 76	1098	279	N/A	1000
Subsampled	80.00	10	40	2.18	6472	19.54	974	103	1000	780
Subsampled	80.00	10	40	2.04	7127	16.05	989	112	1000	900
Subsampled	80.00	10	40	2.88	4491	17.33	926	80	1000	1000
Subsampled	72.00	10	40	1.92	7057	17.42	983	108	900	900
Subsampled	70.00	10	40	2.96	4409	20.24	924	80	1000	1000
Subsampled	70.00	15	40	2.96	4449	17.70	932	74	1000	1000
Subsampled	70.00	15	45	2.80	4779	21.76	939	73	1000	1000
Subsampled	70.00	10	40	1.87	7102	17.44	983	123	1000	900
Subsampled	63.00	10	40	2.78	4416	21.27	921	76	900	1000
Subsampled	63.00	10	40	1.92	7087	16.20	984	114	900	900
Subsampled	54.60	10	40	1.74	7045	19.41	977	126	780	900
Subsampled	54.60	10	40	2.02	6398	14.52	963	110	780	780

Estimate genome size-s indicates the value of genome size used as parameter for subsampling PacBio reads from the raw data
Estimated genome size-a indicates the value of genome size used as parameter in the WTDBG2 assembler

Supplementary Material and Methods

Specimens, tissue and blood sampling, and agarose embedding of red blood cells

Specimens of the Antarctic notothenioid fish *Trematomus borchgrevinki* were caught from McMurdo Sound (77.5°S, 165°E), Antarctica by hook and line through holes drilled through annual sea ice and transported back to the aquarium facility at McMurdo Station. Fish were anesthetized using MS222 (Sigma) and blood was drawn from the caudal vein using needle and syringe and expressed into collection tubes containing heparin (final concentration 250 U/mL). The fish was then sacrificed, and various tissues were dissected and preserved in -20°C pre-chilled 90% ethanol, and stored at -20°C, or flash frozen in liquid nitrogen and stored at -80°C. All fish handling and sampling complied with the University of Illinois, Urbana-Champaign (UIUC) IACUC approved protocol.

The heparinized red blood cells (RBCs) were embedded in agarose as follows. RBCs were first gently spun down and washed with notothenioid PBS (phosphate buffered saline, 500 mOsm, pH 8.4). Aliquots of washed RBCs of known concentration (determined with a hemocytometer) were embedded in 1% low melting point agarose plugs (1 cm×0.5 cm×0.75 cm) using BioRad plug molds to prevent shearing of high molecular weight (HMW) genomic DNA, following Miyake and Amemiya (2004). Each plug contained an appropriate number of RBCs to provide about 20 µg of DNA, based on an estimated 1C genome size of 1.1 pg. The agarose embedded RBCs were then lysed exhaustively *in situ* using a lysis buffer (1% lithium dodecyl sulfate, 10mM Tris-HCl pH 8.0, 100mM EDTA, pH 8.0), and preserved in a preservation buffer (0.2% N-laurylsarcosyl, 2mM Tris-HCl, 100mM EDTA, pH 9.0). The preserved agarose plugs were returned to UIUC for DNA extraction.

High molecular weight (HMW) genomic DNA preparation

HMW DNA was prepared for sequencing on three different platforms – Illumina, Oxford Nanopore, and Pacific Biosciences (PacBio) Sequel II, using the agarose embedded RBC and/or muscle tissue. Agarose blocks of embedded RBC DNA derived from a single male *T. borchgrevinki* were thoroughly desalted by equilibration with 0.5x TE (5mM Tris-HCl, 0.5mM EDTA, pH 8.0) at 4°C with three buffer changes, followed by equilibration with 1x β-agarase buffer (10 mM Bis-Tris, 1 mM EDTA, pH 6.5). Individual plugs were then heated at 65°C for 15 minutes to melt the agarose, then cooled to 42°C. Two units of β-Agarase I (New England BioLabs) was gently stirred into each melted agarose plug, and the sample was incubated at 42°C for 1-2 hour. The digested (liquified) plug was then incubated with proteinase K (final concentration of 2 mg/mL) at 55°C for one hour.

For Illumina and Nanopore sequencing of the male *T. borchgrevinki*, the DNA was recovered from the digested plug by one gentle extraction with phenol:chloroform (1:1), transferring the DNA-containing aqueous phase into Spectra/Por 3 dialysis tubing (MW cutoff 3,500 Da), followed by dialyzing exhaustively against 0.5x TE. For PacBio CLR (continuous long read) sequencing, the DNA was recovered from the digest using

the SPRI paramagnetic bead-based GenFind v.3 kit (Beckman Coulter) following vendor instructions, but with two additional DNA elutions (for a total of three). HMW DNA from pectoral muscle of the same male fish was additionally isolated for Nanopore sequencing. Muscle was finely minced and digested with proteinase K (final concentration 400μg/mL) in a lysis buffer (10 mM Tris, 100 mM EDTA, 1% N-lauryl sarcosine, 0.2% sodium deoxycholate, pH 8.0) at 55°C for 4 hours, followed by RNaseA digest (final concentration 200μg/mL) at 40°C for 2 hours. The digest was gently extracted once with phenol:chloroform (1:1), and the DNA in the aqueous phase was precipitated with isopropanol, washed with 70% ethanol, dried, and resolubilized in 0.5x TE. For PacBio CLR sequencing of a second *T. borchgrevinki* individual (a female), HMW DNA was isolated from frozen white muscle using the Nanobind Tissue Big DNA kit (Circulomics) following vendor instructions.

The concentrations of recovered HMW DNA were determined using Qubit dsDNA Broad Range Assays and Qubit v.3 fluorometer (Invitrogen). The integrity and MW range of the DNA were assessed by pulsed-field electrophoresis using a BioRad CHEF Mapper XA system, and Fragment Analyzer (Advanced Analytical). The DNA were of high purity and integrity, and achieved MW of 35kp to ≥150Kbp with the phenol:chloroform extraction method, and 48Kbp to ≥190Kbp with GenFind v.3 and Nanobind Tissue Big DNA kit, with insignificant fraction below the lower bound.

CONTEX algorithm

The CONTEX Python program parses the comma-separated values file generated by INFO so that information related to each fragmented BUSCO gene is extracted one at a time. If a gene was found as *complete* in a corresponding, reverse complemented scaffold the gene is not processed further. For the remaining genes, the orientation of the two scaffolds – one with the *fragmented* gene and one with the *complete* gene – are determined relative to each other by examining the respective flanking gene(s) on one or both side(s). If the comparison shows that the order of the flanking gene(s) along the two scaffolds is consistently the same or opposite, the analysis continues. If the relative order of the adjacent gene(s) is inconsistent or if there is overlap between the complete or fragmented gene and neighboring gene(s), then the direction of the scaffold cannot be determined and the gene is filtered out from the downstream analysis.

If the scaffold containing the complete and/or fragmented gene contains only one gene the orientation is determined by CONTEX using a two-step process, however, the second step is only performed when the first step is unsuccessful. In the first step, k-mers of flanking sequences from one or both sides of the complete BUSCO gene, depending on start and end positions of the gene, are searched against unique sets of k-mers generated independently from the scaffold and its reverse complement. The direction of the scaffolds containing the complete and fragmented genes are considered to be in the same or opposite orientations if the flanking sequences match only to the k-mers from the forward or reverse-complemented scaffold, respectively. If the flanking sequences map to k-mers from both the forward and reverse complemented scaffold or from none of them, CONTEX maps the k-mers of the whole scaffold containing the complete BUSCO gene to the k-mers of the whole scaffold containing the fragmented

BUSCO gene. CONTEX implements a user defined percentage of shared k-mers between the scaffolds to define the relative direction of scaffolds.

After determining the orientation of the scaffolds, CONTEX k-merizes the scaffold containing the fragmented gene and retains non-repetitive and non-palindromic k-mers. CONTEX also k-merizes the flanking sequences from the scaffold containing the complete BUSCO gene. The k-mers are matched between the two scaffolds and the contig(s) with the fragmented BUSCO genes are replaced with the contig(s) containing the complete BUSCO gene.

Overview of Quickmerge algorithm

When a specific query contig is aligned to the reference by the `nucmer` component of Mummer, the matching sequence segments of the query are aligned in a linear fashion if the sequences of the query and reference share high nucleotide sequence identity. However, if the query sequence is repetitive, then the alignment order of the query sequence blocks can be disrupted. Regardless, the summation of all the lengths of all aligned and overlapped blocks of the specific query contig to specific reference contig provides the total length of the alignment (i.e., overlapped and aligned (OVL) portion of the query) for that query sequence. Apart from OVL portion, the query contig may contain sequence that overlaps the reference but does not align (n-OVL) as well as sequence that neither overlaps nor aligns (overhang). When delta-filter is employed, it removes alignment blocks below a minimum identity and length. Quickmerge takes the alignment information to calculate the ratio of OVL to n-OVL and to determine any overhangs of the alignment. It considers merging the reference and query contigs based on the OVL/n-OVL ratio: any alignment with a ratio less than 1.5 is not considered for merging. If it merges the contigs, any overhang of the reference and/or query are included in the final product. The OVL of the reference sequence gets priority over the OVL of query while merging.
